# CDC25B partners with PP2A to induce AMPK activation and tumor suppression in triple negative breast cancer

**DOI:** 10.1093/narcan/zcaa039

**Published:** 2020-12-21

**Authors:** Junmei Cairns, Reynold C Ly, Nifang Niu, Krishna R Kalari, Erin E Carlson, Liewei Wang

**Affiliations:** Division of Clinical Pharmacology, Department of Molecular Pharmacology and Experimental Therapeutics, Mayo Clinic, Rochester, MN 55905, USA; Division of Clinical Pharmacology, Department of Molecular Pharmacology and Experimental Therapeutics, Mayo Clinic, Rochester, MN 55905, USA; Division of Clinical Pharmacology, Indiana University School of Medicine, Indianapolis, IN 46202, USA; Division of Clinical Pharmacology, Department of Molecular Pharmacology and Experimental Therapeutics, Mayo Clinic, Rochester, MN 55905, USA; Division of Biostatistics and Informatics, Department of Health Sciences Research, Mayo Clinic, Rochester, MN 55905, USA; Division of Biostatistics and Informatics, Department of Health Sciences Research, Mayo Clinic, Rochester, MN 55905, USA; Division of Clinical Pharmacology, Department of Molecular Pharmacology and Experimental Therapeutics, Mayo Clinic, Rochester, MN 55905, USA

## Abstract

Cell division cycle 25 (CDC25) dual specificity phosphatases positively regulate the cell cycle by activating cyclin-dependent kinase/cyclin complexes. Here, we demonstrate that in addition to its role in cell cycle regulation, CDC25B functions as a regulator of protein phosphatase 2A (PP2A), a major cellular Ser/Thr phosphatase, through its direct interaction with PP2A catalytic subunit. Importantly, CDC25B alters the regulation of AMP-activated protein kinase signaling (AMPK) by PP2A, increasing AMPK activity by inhibiting PP2A to dephosphorylate AMPK. CDC25B depletion leads to metformin resistance by inhibiting metformin-induced AMPK activation. Furthermore, dual inhibition of CDC25B and PP2A further inhibits growth of 3D organoids isolated from patient derived xenograft model of breast cancer compared to CDC25B inhibition alone. Our study identifies CDC25B as a regulator of PP2A, and uncovers a mechanism of controlling the activity of a key energy metabolism marker, AMPK.

## INTRODUCTION

The cell division cycle 25 (CDC25) family of proteins is highly conserved, dual-specific tyrosine phosphatases responsible for regulating cell cycle transition (1,2). This family is responsible for maintenance of normal cell cycle progression and has a role in DNA damage response (1,2), tumorigenesis and drug response (3,4). There are three CDC25 family members (CDC25A, B and C) in mammals, all of which have been implicated in the control of G1/S, S-phase, G2/M transition and mitosis (1,5). The structure of CDC25 proteins can be divided into two main regions: the N-terminal region and the C-terminal region. The N-terminal region is extremely divergent and has sites for its phosphorylation and ubiquitination that regulate the phosphatase activity. The C-terminal region is very conserved and contains the catalytic site (6). In accordance with their critical roles in cell cycle regulation, CDC25A and CDC25B have been shown to be involved in cancer progression. CDC25B has been found to be overexpressed in many primary tumors, including breast cancer (7). As CDC25B promotes cell cycle progression (8) and is overexpressed in numerous rapidly dividing cancer cells, one might expect a correlation between CDC25B overexpression and the rate of proliferation. However, no significant correlation has been observed in many cancers (9–11). Thus, the role of CDC25B in cancer might be more complicated than merely promoting cell cycle progression. It is likely that CDC25B has additional functions beyond its role in Cyclin/CDK activation.

Here, we found that CDC25B interacts with protein phosphatase 2A (PP2A), the major Ser/Thr phosphatase in cells (12). A majority of the soluble phosphatases activity at phospho serine and phospho threonine is catalyzed by PP2A. PP2A exists in a trimeric holoenzyme complex, which consists of three subunits: catalytic (PP2A-C), scaffold (PP2A-A) and regulatory subunits (PP2A-B) (13). PP2A-C exists in two isoforms Cα and Cβ. Both isoforms consist of 309 amino acids and share 97% sequence similarity. Cα is expressed in higher abundance than Cβ (14). The PP2A scaffold subunit, acting as a structural assembly base to escort the catalytic subunit and to facilitate interaction with the regulatory subunit and other substrates, also exists in two isoforms, Aα and Aβ. Both are ubiquitously expressed and share 86% sequence similarity (15). In about 90% of the PP2A assemblies, the holoenzyme is composed of the Aα scaffold subunit that is highly abundant in all normal tissues, while Aβ is found only in 10% of PP2A assembly. The PP2A regulatory subunit is structurally diverse and has a minimum of 26 different transcripts and splice variants in human. PP2A-B subunits are multiforms and are classified into four different families: B55/PR55, B56/PR61, PR48/PR72/PR130 and PR93/PR110. B55 has four different isoforms (α, β, γ and δ). B56 has five different isoforms (α, β, γ, δ and ϵ), which show 80% sequence identity in their central region but differ in their N and C terminals, leading to different expression levels in tissues. Intracellular localization of B56 isoforms varies, as B56γ is expressed in the nucleus, B56α, B56β and B56ϵ are expressed in the cytoplasm, while B56δ appears to be expressed in both the nucleus and cytoplasm (16). One of the known PP2A targets is the adenosine monophosphate (AMP)-activated protein kinase signaling (AMPK) (17).

AMPK is a heterotrimeric kinase consisting of alpha, beta and gamma subunits. AMPK is well known for its role in the regulation and maintenance of cellular metabolism and energy homoeostasis (18). AMPK activation can result in increased activation of anabolic reactions and decreased activation in catabolic reactions. Additional outcomes of AMPK activation include decreased protein synthesis, cell growth, cell cycle arrest, cell death and increased autophagy (19–23). Alterations in AMP/adenosine triphosphate (ATP) ratio allows for AMP/ADP binding to AMPK facilitating a conformation change which in turn activates the AMPK catalytic site on the alpha subunit by phosphorylation from upstream AMPK kinases such as Liver Kinase B1 (LKB1) and calmodulin-dependent protein kinase kinase-β (CaMKKβ) (24). AMPK is deactivated by PP2A-mediated de-phosphorylation of the catalytic site when there is a high ratio of ATP to adenosine diphosphate (ADP) or AMP (17–18,25). It has previously been suggested that the upstream kinases of AMPK are frequently mutated and deleted in various human cancers, including breast cancer (26,27), resulting in reduced AMPK activity and leading to cancer cell growth (28,29). AMPK is therefore a critical target in cancer therapy. It has also been previously suggested that several AMPK activators may suppress cancer cell growth (30–32).

In this study, we demonstrate that CDC25B can regulate AMPK activation through its interaction with PP2A, and block PP2A access to AMPK, resulting in increased AMPK phosphorylation in triple negative breast cancer. CDC25B gene expression is associated with metformin anticancer response in triple negative breast cancer through its regulation of AMPK via PP2A. CDC25B depletion leads to metformin resistance and PP2A inhibition overcomes the resistance to metformin in breast cancer patient derived organoid models, suggesting that CDC25B–PP2A–AMPK axis is an important regulator of breast cancer growth, AMPK activation and metformin response.

## MATERIALS AND METHODS

### Cell culture, antibodies and chemicals

HEK-293T, MCF7, SKBR3, BT549 and HS578T human breast cancer cells were obtained from the American Type Culture Collection (Manassas, VA, USA). AMPK wild-type (WT) and null mouse embryo fibroblast (MEF) cell lines was a gift from Dr Zhenkun Lou (Mayo Clinic). MCF7 and HS578T cells were cultured in Dulbecco's-modified Eagle's medium (DMEM) containing 10% fetal bovine serum (FBS). SKBR3 and BT549 cells were cultured in Roswell Park Memorial Institute (RPMI) 1640 medium containing 10% FBS. HEK-293T cells were cultured in DMEM medium containing 10% FBS. AMPK null and WT MEFs were cultured in DMEM.

Metformin was purchased from Millipore-Sigma. Drugs were dissolved in 1× sterile phosphate-buffered saline (PBS), and aliquots of stock solutions were frozen at −80°C and stored at 4°C when regularly used. LB100 and 2-Deoxy-D-glucose (2-DG) were purchased from Selleckchem. Oligomycin was purchased from Cell Signaling. A769662 was purchased from Abcam.

The following antibodies used for western blot analysis were purchased from Cell Signaling Technologies: CDC25B (9525S), AMPKα (2532S), Phospho-AMPKα (40H9, 2535S), ACC1 (C83B10, 3676S), p-ACC1 (3661S), PP2A subunit A (2039S), PPP2R5D (H5D12, 5687S), PP2A subunit C (2038S), LKB1 (3050), β-actin (4970), CDK1 (9116), RAPTOR (E6O3A, 48648), p-RAPTOR (E4V6C, 89146), ULK1 (D9D7, 6439), p-ULK1 (37762) and GAPDH (5174). pPP2A-C (Y307), and Flag (B3111) antibodies were purchased from MilliporeSigma (SAB4503975). B55δ antibody was purchased from Thermo Fisher Scientific (PA5–30763). CaMKKβ (sc-271674), and CDC25A (F-6, sc-7389) antibodies were purchased from Santa Cruz. The following antibodies were used for immunoprecipitation: Santa Cruz Antibody CDC25B (3F116, sc-70825) and Cell signaling technology PP2A-C (2038S).

### Quantitative reverse-transcription PCR (qRT-PCR)

Total RNA was isolated from cultured cells with the QIAGEN RNeasy kit (QIAGEN Inc., Valencia, CA, USA), followed by quantitative reverse-transcription polymerase chain reaction (qRT-PCR) performed with the one-step Brilliant SYBR Green qRT-PCR master mix kit (Stratagene, La Jolla, CA, USA). Specifically, primers purchased from QIAGEN were used to perform qRT-PCR using the Stratagene Mx3005P real-time PCR detection system (Stratagene). All experiments were performed in triplicate with GAPDH or β-actin as an internal control.

### Transient transfection and RNA interference

Specific siGENOME siRNA SMARTpool reagents against CDC25B, CDC25A, AMPK α1, AMPK α2, CDK1, PPP2CA, PRKAA1, PRKAA2, PPP2R5D and non-targeting siRNA #2 for negative control were purchased from Dharmacon Inc. (Lafayette, CO, USA). Lipofectamine RNAiMAX transfection reagent (Invitrogen, Carlsbad, CA, USA) was used for siRNA transfection. Specifically, cells were seeded into 96-well plates or 6-well plates and mixed with siRNA-complex consisting of 20–50 nmol of specific siGENOME siRNA SMARTpool or non-targeting negative control (Dharmacon) and Lipofectamine RNAiMAX transfection reagent.

CDC25B plasmid was purchased from Open Biosystems and then subcloned into the pIRES-2-EGFP plasmid vector. Sequence was verified by Sanger sequence for both strands. pCMV-HA-AMPKα1, pCMV-HA-PP2A-Cα were gifts from Dr Zhenkun Lou (Mayo Clinic). CDC25A plasmid was purchased from Genscript. Plasmids used in downstream experiments were transfected with Lipofectamine 2000 (Invitrogen, Carlsbad, CA, USA).

### Immunoblotting

Cells were harvested and lysed using 1× NETN lysis buffer containing protease (cOmplete, ethylenediaminetetraacetic acid (EDTA)-free, Millipore Sigma) and phosphatase inhibitors (PhosSTOP, Millipore Sigma) on ice for 30 min followed by microcentrifugation at 4°C for 15 min at 12 000 rpm. Cell lysates were isolated and quantified for protein concentration and then boiled in 4× sodium dodecyl sulfate (SDS)-loading buffer for 5 min at 100°C. Cell lysates were subjected to SDS-polyacrylamide gel electrophoresis (SDS-PAGE) followed by semi-dry transfer onto polyvinylidene fluoride (PVDF) membranes. PVDF membranes were blocked using 5% non-fat milk in 1× TBST solution for 1 h at room temperature. Membranes were incubated with primary antibodies diluted in 5% milk 1× TBST overnight in 4°C. Following overnight incubation, membranes were washed with 1× TBST three times for 5 min each and then incubated with secondary antibodies diluted in 5% milk 1× TBST for 1 h at room temperature. Membranes were then washed with 1× TBST three times for 5 min. Protein bands were visualized using chemoluminescence (Thermo Scientific, Rockford, IL, USA).

CDC25B was immunoprecipitated from HS578T cells and performed mass spectrometry to identify CDC25B interacting proteins.

### Co-immunoprecipitation and silver staining

Cell extract from HS578T were prepared using a lysis buffer consisting of 50 mM HEPES, (pH 7.5), 150 mM NaCl, 1 mM EDTA, 2.5 mM ethylene glycol tetraacetic acid (EGTA), 10% glycerol, 0.1% Tween-20, 1 mM dithiothreitol (DTT), 1 mM NaF, 0.1 mM Na3VO4, 10 μg/ml leupeptin, 2 μg/ml Aprotinin and 0.1 mM phenylmethanesulfonyl fluoride (PMSF). Briefly, the extract were sonicated for 10 s and then centrifuged at 14 000 rpm at 4°C for 15 min. Equal amount of total protein from the extract were incubated with 2 μg of CDC25B (Santa Cruz, sc-70825) overnight at 4°C on a rotator. Protein A-conjugated agarose beads (Pierce Protein A Agarose, 20 333) (30 μl) were then added and incubated for 1 h on a rotator at 4°C. The beads were pelleted at 4000 rpm at 4°C and then were washed five times in 500 μl lysis buffer. Precipitated proteins were then dissolved in SDS-sample buffer and separated by SDS-PAGE for silver staining.

Proteins were separated on Biorad precast polyacrylamide gel (Cat#4561084). Gels were stained with the Silver Stain Kit for Mass Spectrometry (Thermo Scientific Pierce, PI24600) following the vendor's protocol. Protein bands were cut with a sterilized blade and detained with the same kit. Samples were sent to the Taplin Mass Spectrometry facility at Harvard Medical School for analysis of potential interacting proteins. The mass spectrometry data have been deposited in a repository affiliated with the ProteomeX consortium (accession: PXD020368).

### Co-immunoprecipitation

Cells were lysed with 1x NETN buffer (20 mM Tris–HCl, pH 8.0, 100 mM NaCl, 1 mM EDTA, 0.5% Nonidet P‐40) containing protease (cOmplete, EDTA-free, Millipore Sigma) and phosphatase inhibitors (PhosSTOP, Millipore Sigma) on ice for 25 min. After centrifugation, cell lysates were incubated with 2 μg antibody and protein A or G sepharose beads (Amersham Biosciences) for 3 h at 4°C. The immunocomplexes were then washed with NETN buffer for four times, and the immune-complexes were separated by SDS-PAGE. Immunoblotting was performed following standard procedures.

### Protein expression and purification

Recombinant Human CDC25B and CDC25A protein, fused to GST-tag, was expressed in *Escherichia coli* and purified by GSH-sepharose. Recombinant Human PP2A-A, PP2A-B and PP2A-C, fused with a polyhistide tag at the N-terminus, was produced in *E. coli*. (Creative Biomart). The GST-CDC25B plasmids were transformed into *E. coli* cells. Overnight cultures of *E. coli* containing CDC25B expression constructs were used to inoculate 2?YT medium containing ampicillin (100 μg/ml), grown until the OD600 reached 0.6–0.8, and then induced with 0.5 mM Isopropyl-β-D-thiogalactoside (IPTG) overnight at room temperature. Cells were harvested, lysed by sonication in 50 mM Tris (pH 8.0), 250 mM NaCl, 5% glycerol and 1 mM DTT, and purified on glutathione resin. Precession protease was used to release CDC25B proteins from the resin, followed by further purification on a SuperoseTM 6 (GE Healthcare) column.

### GST pull-down experiments

For GST pull-down experiments, GST-CDC25B, His-PP2A-A, His-PP2A-B and His-PP2A-C subunits were expressed and purified in *E. coli*. Equal amounts of different subunits were mixed to form the PP2A subcomplex AB, AC, BC and holoenzyme ABC. His-PP2A-A, B, C, A + B, A + C, B + C or A + B + C (2 μg each subunit) was mixed with 3 μg of GST-CDC25B protein purified from *E*. *coli*, andincubated with the glutathione resin in 50 mM HEPES (pH 6.9), 50 mM NaCl, 4 mM cysteine and 0.2% dimethyl sulfoxide (DMSO). After washing five times with 50 mM HEPES (pH 6.9), 50 mM NaCl and 1 mM DTT, 1× SDS-PAGE loading dye was added to the glutathione resin and the pull down was analyzed by SDS-PAGE.

### Cytotoxicity and cell growth assays

Cytotoxicity assays were performed in triplicate at each drug concentration. Cells were seeded in 96-well plates and mixed with siRNA-complex consisting of 20–50 nmol of specific siGENOME siRNA SMARTpool or non-targeting negative control (Dharmacon) and Lipofectamine RNAiMAX transfection reagent. After 24 h, cells were treated with 10 μl of metformin at final concentrations of 0, 0.78, 1.56, 3.125, 6.25, 12.5, 25, 50 and 100 mM for additional 48–72 h. Cytotoxicity was measured using CYQUANT assay (#C35012, Invitrogen) following the manufacturer's instructions.

Cell growth assays were performed in triplicate. Specifically, 2000 cells/well were plated in 96-well plates, followed by different treatments depending on the experiments. CYQUANT Direct Cell Proliferation Assay kit (#C35012, Invitrogen) was used to determine the cell viability, following the manufacturer's instructions. Briefly, 100 μl of CYQUANT assay solution was added, and plates were incubated at 37°C for 1 h, and then read in an Infinite M1000 Pro plate reader (Tecan AG, Switzerland) with filters appropriate for 480 nm excitation and 520 nm emission. Cell proliferation was monitored every 2 days.

### CDC25B knockout and stable knockdown

CRISPR Nickase was used to create CDC25B knockout BT549 cell line. CDC25B double nickase plasmid (h) (sc-401457-NIC), CDC25B double nickase plasmid (h2) (sc-401457-NIC-2) and control double nickase plasmid (sc-437281) were purchased from Santa Cruz. 1.5 × 10^5^ − 2.5 × 10^5^ cells were seeded in a 6-well plate. A total of 3 μg of plasmid DNA were transfected with Lipofectamine 2000 (Invitrogen). GFP-positive cells were screened to select for successfully transfected cells in growth medium containing puromycin at 1–2 μg/ml for 2 weeks.

CDC25B stable knockdown HS578T cell line was created using five different TRC CDC25B shRNAs plasmids in pLKO.1 lentiviral vector (GE HEALTHCARE). Only shRNA four and five plasmids were successfully transfected in HS578T and selected. pLKO.1 control vector plasmid was a gift from Dr Taro Hitosugi (Mayo Clinic). Lentiviral shRNA, pHRCMV8.2ΔR and CMV-VSVG plasmids were co-transfected into 293T cells for 48 h using Lipofectamine 2000 to generate lentiviral soup. Following 48 h, supernatant containing lentivirus was isolated then used with 8 μg/ml polybrene to infect subconfluent HS578T cells. Cells were selected in growth medium containing 1–2 μg/ml of puromycin for 2 weeks.

### CDC25B subcloning

CDC25B N-terminal and C-terminal truncated cDNAs were generated with PCR using Q5 Hot Start High-Fidelity DNA Polymerase (New England Biolabs) and the following forward and reverse primers for N and C-terminal truncated CDC25B cDNAs. CDC25B truncated fragments were cloned into pIRES-2-EGFP vector using SalI and BamHI restriction enzymes.

1–375: Forward primer: 5′-ACGCGTCGACATGGAGGTGCCCCAGCCG-3′, Reverse primer: 5′-CGGGATCCTGAGCGGAGGACGCGGGC-3′. 374–580: Forward primer 5′-ACGCGTCGACAAATCACTGTGTCACGAT-3′, Reverse primer 5′- CGGGATCCTCACTGGTCCTGCAGCCG-3′.

CDC25B mutation clones C488S, S353A and S353E were generated via site-directed mutagenesis using Quikchange Lighting site-directed mutagenesis kit (Stratagene) in pIRES-2-EGFP vector. Sequence was verified by Sanger sequence for both strands.

### Cell cycle assay

Cells were plated in 60-mm dishes, and transfected with negative siRNA or CDC25B siRNA. Forty-eight hours later, cells were harvested by trypsinization. After fixing, cells were stained with propidium iodide, and analyzed by a flow cytometer (BD FACScans). Three independent experiments were performed and at least 20 000 cells were counted, the proportions of cells in different cell cycle phases were gated and calculated using the software Flowjo 8.7.1 (Tree Star, Inc.).

### Immunofluorescence

Cells were plated in 16-well cover-glass slips (Thermo Fisher, C37000) and were transfected with CDC25B constructs. Cells were then treated with 10 mM metformin for 2 days and were washed with 1 × PBS before being fixed with 4% paraformaldehyde for 10 min, followed by permeabilization using 0.1% Triton X-100. Cells were then blocked with 3% bovine serum albumin for 30 min and were incubated with specific primary antibody overnight at 4°C. They were then incubated with the appropriate Alexa Fluor antibody for 1 h at room temperature. The cells were stained with DAPI before visualization using Zeiss LSM 780 Confocal Microscope at 100× magnification.

### Thr and Tyr phosphatase activity assays

Three hundred micrometer of pThr peptide (KRpTIRR, Milipore), pSer peptide (RRApSVA, Milipore) or pH2AX peptide (KATQASQEpY, Abgent) were incubated with 200–500 ng of PP2A protein in a 25 μl phosphatase reaction. An equal amount of purified protein was used in each set of phosphatase reaction. The reaction was incubated at 37°C for 1 h after which 100 μl Malachite Green mixture was added and allowed to incubate for 15 min. Absorbance was measured at 620 nm using an Infinite M1000 Pro plate reader. All phosphatase activity assays were normalized to the amount of protein used.

### PP2A activity assay

Cells (1  ×  10^6^ cells) were lysed using NP-40 lysis buffer. PP2A activity was measured using a PP2A Immunoprecipitation Phosphatase Assay Kit (EMD Millipore). Briefly, protein lysates were incubated with PP2A antibody at 4°C with continuous rotation for 2 h. Following the addition of assay buffers and malachite green solution, the plate was read at an absorbance of 650 nm using a microplate reader (Infinite M1000 Pro). Phosphatase activity was determined using a standard curve. Experiments were repeated at least in triplicate, and phosphatase activity was reported as mean fold change ± SEM from the untreated sample.

### Patient-derived xenografts generation and organoid isolation, 3D cell culture and growth assay

Breast cancer patient-derived xenografts (PDXs) from the Breast Cancer Genome Guided Therapy Study (BEAUTY) (NCT02022202) were generated according to previously described protocol (33). All patients provided written informed consent. All procedures of animal studies were performed according to the National Institutes of Health guideline with approval obtained from the Mayo Clinic Institutional Animal Care and Use Committee (IACUC) and Biosafety Committee. Female non-obese diabetic (NOD)—Cg-Prkdcscid Il2rgtm1Wjl/SzJ (NSG) mice (six to eight weeks) from Jackson Laboratories (Bar Harbor, ME, USA) were used. Percutaneous breast cancer biopsies obtained prior to neoadjuvant chemotherapy in patients were used for xenograft establishment (33). Mice were killed by CO_2_ inhalation once the tumor size met the IACUC guideline.

Tumor cells from breast cancer PDXs were isolated using the human tumor dissociation kit (Miltenyi Biotec, San Diego, CA, USA). Briefly, tumors were minced into small pieces of 2–4 mm, and then transferred into the gentle MACS C tube and run the 7C_h_TDK3 program according to manufacturer's protocol. The tubes were then centrifuged to collect the sample material. The cell suspension was then applied to a MACS SmartStrainer (70 μm). Mouse Cell Depletion Kit (Miltenyi Biotec) was used to enrich human cells. Specifically, cell pellet was suspended in buffer, and 20 μl of the Mouse Cell Depletion Cocktail was added and incubated for 15 min at 4°C. Then magnetic separation with LS Columns was performed to collect human cells. Human tumor cells were plated in 96-well low binding NanoCulture plate (Organogenix) (10^4^ cells/well) in DMEM supplemented with 10% fetal calf serum (FCS), 1% glutamax (Life Technologies), 1% sodium pyruvate (Life Technologies), non-essential amino acids (Life Technologies) and 1% Penicillin-Streptomycin (Life Technologies) and cultured at 37°C, 5% CO_2_. After 3 days, 5 μM of ROCK inhibitor (Tocris Bioscience) was added to the culture medium for a week. The organoids were then cultured in medium without ROCK inhibitor for 3 days.

Organoids were transfected with siCDC25B, Flag-CDC25B, Flag-CDC25B C488S or Flag-CDC25B S353E using Lipofectamine 2000 (Invitrogen). Organoids survival assays were performed in three replicates at each treatment. After optimization, 4–100 mM doses of metformin were chosen for metformin response assay in organoids to have a broad range of coverage. A total of 20 mM of metformin efficiently stimulated AMPK in organoids and was therefore used in the subsequent growth assay experiments. Specifically, organoids were treated with 0, 4, 20 and 100 mM of metformin for 3 days, and survival was measured using the luminescent CellTiter-Glo Viability assay (Promega). A total of 100 μl of CellTiter-Glo reagent was added into each well, followed by mixing contents for 2 min on an orbital shaker to induce cell lysis. Plates were incubated at 37°C for 1 h, and the luminescent signal was measured using an Infinite M1000 Pro plate reader. Organoids growth assays were performed in triplicate, and growth was monitored every 2 days. Organoids were lysed with SDS buffer and subjected to western blot.

### Statistical analysis

All data were generated with a minimum of three technical replicates. For cell survival, cell proliferation, gene expression and quantifications, data are represented as the mean ± SEM of three independent experiments. Unless otherwise described, 2-way ANOVA (Analysis of Variance) was performed to test group difference. Then post-hoc analysis was carried out to check if specific groups are significantly different or similar. Tukey HSD (Tukey Honest Significant Differences, R function: TukeyHSD()), which is essentially a modified t-test corrected for multiple comparisons, was applied in some of the analysis. Statistical significance level is 0.05. *P < 0.05; **P < 0.01.

## RESULTS

### CDC25B regulates AMPK activation

**Figure 1.**
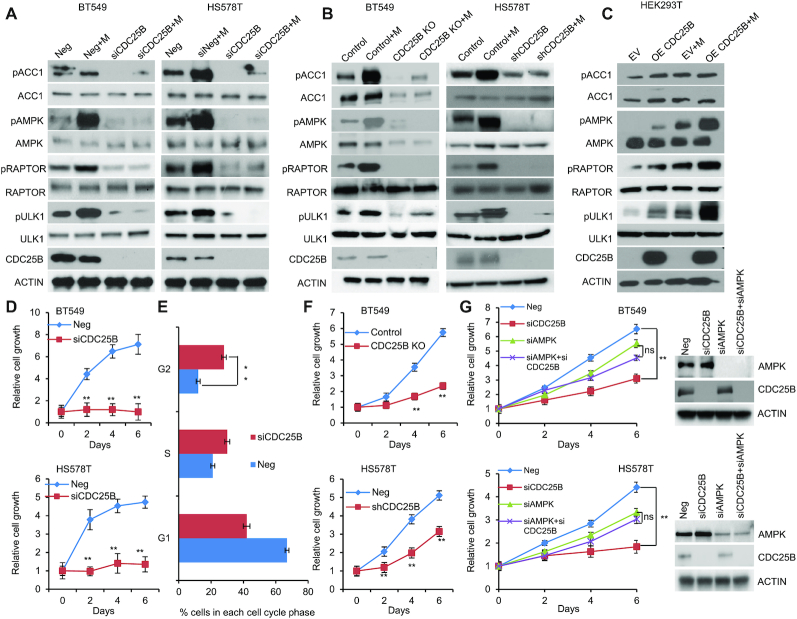
CDC25B suppression inhibits AMPK activation and inhibits cell growth in triple negative breast cancer. (**A**) BT549 and HS578T cells were transfected with negative siRNA (Neg) or CDC25B siRNA for 24 h, and then treated with 20 mM metformin for 48 h. Cells were harvested for immunoblotting of AMPK pathway. (**B**) CDC25B knockout or control BT549 and CDC25B shRNA or control HS578T cells were treated with 20 mM metformin for 48 h. Cell lysates were then blotted with the indicated antibodies. (**C**) HEK-293T cells were transfected with empty vector (EV) or CDC25B plasmids. Twenty-four hours later, cells were treated with 20 mM metformin for additional 48 h. Cell lysates were subjected to western blot. (**D**) Knockdown of CDC25B decreased BT549 and HS578T cell proliferation. Cell proliferation was monitored every 2 days. The *x*-axis indicates time post transfection, and the *y*-axis indicates relative cell growth to day 0. (**E**) Knockdown of CDC25B increased cells in G2/M phase. BT549 cells transfected with negative or CDC25B siRNAs were subjected to flow cytometry. Proportion of cells in each cell cycle was quantified. (**F**) CDC25B knockout or stable knockdown decreased proliferation compared to control BT549 and HS578T cells. Cell proliferation was monitored every 2 days. The *x*-axis indicates time, and the *y*-axis indicates relative cell growth to day 0. (**G**) BT549 and HS578T cell proliferation was monitored every 2 days. Knockdown efficiency was measured by western blot. Data information: all data presented are in the format of mean ± SEM of *N* = 3 independent experiments with three biological replicates for each experiment. For (D and F), statistically significant differences were determined using the two-way ANOVA (**P < 0.01). For (E and G), statistically significant differences were determined using the two-way ANOVA plus Tukey (**P < 0.01), ns represents not significant.

To study the role of CDC25B in patients with breast cancer (7,10,34), AMPK activity was initially evaluated in triple negative breast cancer cells expressing endogenous CDC25B. Breast cancer cells (BT549 and HS578T) showed reduced AMPK phosphorylation at threonine 172 upon CDC25B knockdown (Figure 1A). The effect was confirmed by observing significant reduction of AMPK downstream substrates, ACC1 (S79), RAPTOR (S792) and ULK1 (S317) in CDC25B knockdown cells (Figure 1A). Moreover, CDC25B knockdown dramatically reduced metformin-mediated AMPK phosphorylation in BT549 and HS578T cells (Figure 1A). The regulation of CDC25B on AMPK activity was further confirmed in CDC25B knockout or stably knockdown BT549 and HS578T cell lines, in which CDC25B knockout or stable knockdown reduced AMPK phosphorylation and metformin failed to activate AMPK (Figure 1B). We then overexpressed CDC25B in HEK-293T cells as they have low endogenous CDC25B expression. AMPK phosphorylation was significantly upregulated in CDC25B overexpressed cells compared to control cells, and the effect of CDC25B on AMPK phosphorylation was further enhanced in the presence of metformin (Figure 1C). Furthermore, cells with CDC25B transient knockdown showed slower cell growth compared with control siRNA transfected BT549 and HS578T breast cancer cells (Figure 1D), a process that was through the blocking of cell cycle progression (Figure 1E and Supplementary Figure S1A). CDC25B knockout or stably knockdown cells also grew slower compared with control BT549 and HS578T cells (Figure 1F). We then tested whether AMPK is involved in CDC25B—mediate cell growth regulation. To accomplish this, we knocked down CDC25B in BT549 and HS578T cells with AMPK knockdown. As shown in Figure 1G, CDC25B depletion had minimal effect on the growth of AMPK knockdown cells, indicating CDC25B’s effect on cell growth was at least partially dependent on AMPK. To rule out the possibility that the impact of CDC25B on cell growth is solely mediated by CDK1, a known target of CDC25B, we measured CDC25B effect on cell growth in CDK1 knockdown cells. CDC25B knockdown could still inhibit growth in cells lack of CDK1 (Supplementary Figure S1B), suggesting that CDC25B has additional targets influencing cell growth. These results suggest that CDC25B suppression downregulated AMPK activity and inhibited breast cancer cell growth.

### CDC25B associates with the Ser/Thr phosphatase PP2A

**Figure 2.**
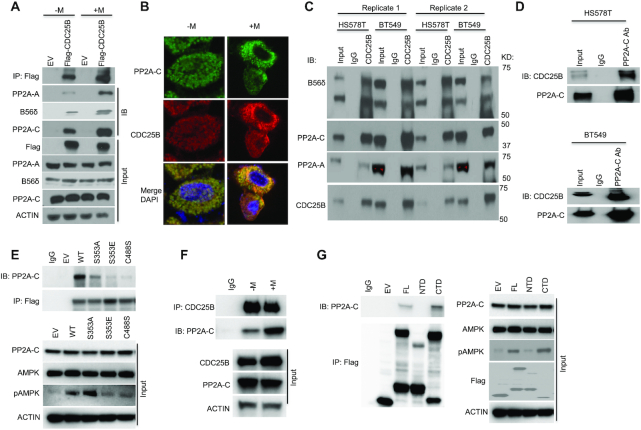
CDC25B interacts with PP2A. (**A**) HEK-293T cells were transfected with EV or Flag-CDC25B plasmids for 24 h then treated with 10 mM metformin for 48 h. Cell lysates were subjected to immunoprecipitation with anti-Flag antibody. The immunoprecipitates were blotted with the indicated antibodies. (**B**) HEK-293T cells were treated with 10 mM metformin treatment for 48 h. PP2A-C and CDC25B were detected using immunofluorescence. The nucleus was labeled by DAPI staining. Images were taken at 100× magnification. (**C**) BT549 and HS578T cell lysates were subjected to immunoprecipitation with control IgG or anti-CDC25B antibody. The immunoprecipitates were blotted with the indicated antibodies. (**D**) BT549 and HS578T cell lysates were subjected to immunoprecipitation with control IgG or anti-PP2A-C antibody. The immunoprecipitates were blotted with the indicated antibodies. (**E**) CDC25B knockout BT549 cells were transfected with EV, WT CDC25B (WT), CDC25B S353A, CDC25B S353E or CDC25B C488S constructs for 48 h. Cell lysates were subjected to immunoprecipitation with control IgG or anti-Flag antibody. The immunoprecipitates were blotted with the indicated antibodies. (**F**) BT549 cells were treated with 20 mM metformin for 48 h. Cell lysates were subjected to immunoprecipitation with control IgG or anti-CDC25B antibody. The immunoprecipitates were blotted with the indicated antibodies. (**G**) CDC25B knockout BT549 cells were transfected with EV, FL CDC25B, CDC25B N-terminal domain (1–373 aa, NTD) or CDC25B C-terminal domain (374–580 aa, CTD) for 48 h. Cell lysates were subjected to immunoprecipitation with control IgG or anti-Flag antibody. The immunoprecipitates were blotted with the indicated antibodies. Data information: all data presented are a representation of *N* = 3 independent experiments.

To elucidate the mechanisms of CDC25B on AMPK activation, co-immunoprecipitation (co-IP) coupled with 2D gel electrophoresis followed by liquid chromatography-mass spectrometry of the IP product was conducted to identify CDC25B-interacting proteins in the triple negative breast cancer cell line, HS578T. One of the oligopeptides was identified as PPP2R5D, a B56δ subunit of PP2A, suggesting that CDC25B could regulate AMPK phosphorylation via its interactions with PP2A. We confirmed the interaction between PP2A and CDC25B using immunoprecipitation of Flag-tagged CDC25B from HEK293T cells followed by western blot analysis with anti-PP2A-A, anti- B56δ and anti-PP2A-C antibodies (Figure 2A). We further confirmed the association between CDC25B and PP2A by using the immunofluorescence assay (Figure 2B). Moreover, we demonstrated endogenous association between CDC25B and PP2A in multiple triple negative breast cancer cell lines (Figure 2C and D). We further showed that overexpression of the phosphatase-dead CDC25B C488S (35) or the S353E mutant, which mimic phosphorylation by Aurora A (36), significantly reduced the CDC25B–PP2A interaction (Figure 2E). While the S353A mutant, resulting in a weaker mitotic inducing effect than that of the WT (36), showed less impact on the CDC25B associations with PP2A. This CDC25B S353 phosphorylation led to its localization to the centrosome, facilitating mitotic entry, suggesting that the interaction between CDC25B and PP2A increased when CDC25B was not engaged in mitotic regulation. Indeed, we observed that the CDC25B–PP2A association occurred at G1 and S phase (Supplementary Figure S2A). We then evaluated whether there might be a change in CDC25B–PP2A interaction in cells treated with metformin. We showed that endogenous CDC25B–PP2A interaction increased with metformin treatment (Figure 2F). We next evaluated whether the interaction is dependent on PP2A phosphatase activity. Using immunoprecipitation in triple negative breast cancer cells treated with LB100, a PP2A inhibitor, we demonstrated that LB100 significantly reduced the association between PP2A and CDC25B (Supplementary Figure S2B). To decipher which domain of CDC25B might interact with PP2A, we expressed the N-terminal domain (NTD) (residues 1–375 aa) or the C-terminal domain (CTD) (residues 374–580 aa) of CDC25B in CDC25B-depleted HEK293T cells. IP of full length (FL) CDC25B and the various CDC25B deletion constructs demonstrated that the CTD of CDC25B predominantly interacted with PP2A, while the NTD had little association with PP2A (Figure 2G). These data again support the finding that CDC25B associates with PP2A, and further localize this interaction to its CTD.

### CDC25B directly interacts with the C subunit of PP2A

**Figure 3.**
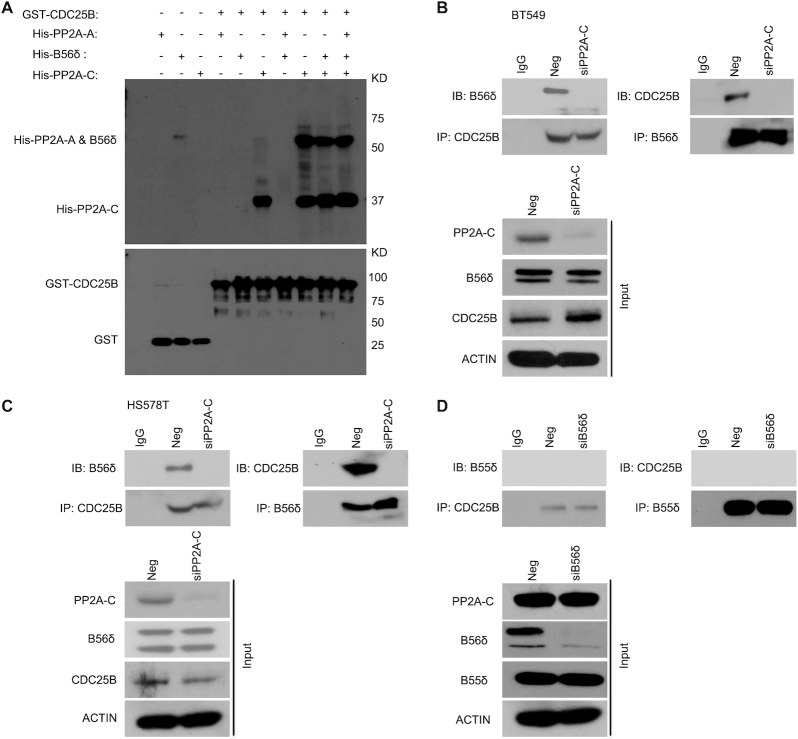
CDC25B interacts directly with the PP2A-C subunit. (**A**) Top: GST-CDC25B was used to pull down purified His-PP2A-A, His-B56δ, His-PP2A-C, His-PP2A-A + B56δ, His-PP2A-A + PP2A-C, His-B56δ + PP2A-C or His-PP2A-A + B56δ + PP2A-C. Bottom: GST immunoblot of SDS-PAGE gel demonstrating the amount of CDC25B used for pull-down experiments. (**B** and**C**) The CDC25B-B56δ interaction in Neg or PP2A-C KD cells. BT549 and HS578T cells were transfected with Neg or siPP2A-C. Forty-eight hours later, cell lysates were subjected to IP with control IgG or anti-CDC25B antibody examining the CDC25B–B56δ interaction. Reciprocal IP with B56δ antibody was also performed to examine the CDC25B–B56δ interaction. The immunoprecipitates were blotted with the indicated antibodies. (**D**) B56δ, not B55δ, interacts with CDC25B. BT549 cells were transfected with siB56δ. Forty-eight hours later, cell lysates were subjected to IP with control IgG or anti-CDC25B antibody examining the CDC25B–B55δ interaction. Reciprocal IP with B55δ antibody was also performed to exam the CDC25B–B55δ interaction. The immunoprecipitates were blotted with the indicated antibodies. Western blot analysis demonstrates the protein level of B55δ in Neg or B56δ KD cells. Data information: all data presented are a representation of *N* = 3 independent experiments.

To further decipher which subunit PP2A might directly interact with CDC25B, we expressed and purified His-PP2A-A, His-B56δ and His-PP2A-C from *E. coli*. The PP2A holoenzyme assembled using these purified proteins had both Ser and Thr phosphatase activities (Supplementary Figure S3), suggesting that these purified subunits were well-folded and fully functional. Using purified proteins, we demonstrated that His-PP2A-C alone, His-PP2A-A + His-PP2A-C, His-B56δ + His-PP2A-C or His-PP2A-A + His-B56δ + His-PP2A-C could pull down purified GST–CDC25B, while His-PP2A-A alone, His-B56δ alone or His-PP2A-A + His-B56δ could not; demonstrating that the C subunit is necessary and sufficient for the interaction with CDC25B (Figure 3A). We further demonstrated that knockdown of the C subunit abolished the association of the B56δ subunit with CDC25B in triple negative breast cancer cells (Figure 3B and C) using anti-CDC25B antibody for IP or anti-B56δ antibody for reciprocal IP. Finally, CDC25B appeared to specifically interact with the B56δ subunit of PP2A, as we could not detect an association between a different B subunit, B55δ, which has been reported to be involved in AMPK regulation (17), upon Negative siRNA (Neg) or B56δ knockdown (siB56δ) (Figure 3D). Taken together, our data demonstrate that CDC25B physically interacts with the PP2A holoenzyme through the C subunit.

### CDC25B–PP2A regulates AMPK activity

**Figure 4.**
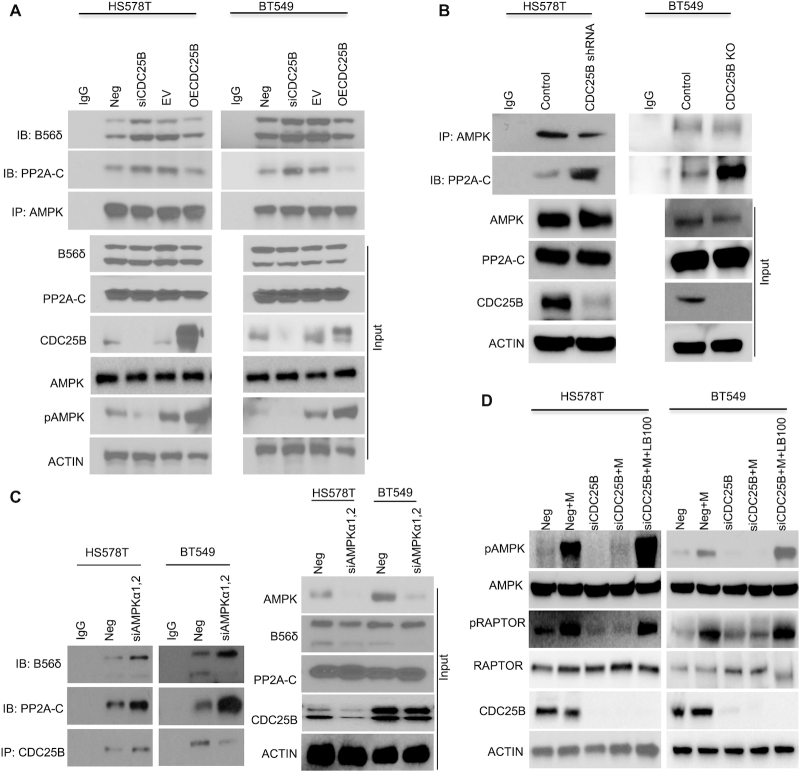
CDC25B alters PP2A interaction with AMPK and subsequent AMPK phosphorylation. (**A**) BT549 and HS578T cells were transfected with Negative siRNA, siCDC25B, EV or CDC25B plasmids. Forty-eight hours later, cell lysates were subjected to immunoprecipitation with control IgG or anti-AMPKα antibody. The immunoprecipitates were blotted with the indicated antibodies. (**B**) CDC25B knockout BT549 and stable knockdown HS578T cell lysates were subjected to IP with control IgG or anti-AMPKα antibody examining the AMPK–PP2A-C interaction. The immunoprecipitates were blotted with the indicated antibodies. (**C**) BT549 and HS578T cells were transfected with Negative siRNA or siAMPK α1and α2. Forty-eight hours later, cell lysates were subjected to IP with control IgG or anti-CDC25B antibody examining the CDC25B–PP2A interaction. The immunoprecipitates were blotted with the indicated antibodies. (**D**) BT549 and HS578T cells were transfected with negative siRNA (Neg) or CDC25B siRNA for 24 h, and then treated with 20 mM metformin and 2 μM LB100 for 48 h. Cells were harvested for immunoblotting of AMPK pathway. Data information: all data presented are a representation of *N* = 3 independent experiments.

We next set out to evaluate how CDC25B regulates AMPK activity via PP2A. Since downregulation of CDC25B reduced AMPK phosphorylation, we hypothesized that either CDC25B regulates upstream kinases of AMPK, such as LKB1 and CaMKKβ, or it might affect the AMPK phosphatase, PP2A and its ability to dephosphorylate AMPK. We performed IP to determine the interaction between CDC25B and LKB1 or CaMKKβ. Interestingly, we did not observe any interactions between CDC25B and either upstream kinase (Supplementary Figure S4A) in CDC25B overexpressed cells. No endogenous associations of CDC25B with LKB1 or CaMKKβ were detected in breast cancer cells (Supplementary Figure S4B). Furthermore, depletion of CDC25B did not change the AMPK‐LKB1 interaction or AMPK‐CaMKKβ interaction (Supplementary Figure S5). Our finding of CDC25B interaction with PP2A (Figure 2) led us to hypothesize that CDC25B’s effect on AMPK phosphorylation is through its effect on AMPK - PP2A interaction (17,18). As shown in Figure 4A, transient CDC25B depletion increased AMPK‐PP2A interaction, while overexpression of CDC25B decreased AMPK–PP2A interaction in HS578T and BT549 cells, suggesting that CDC25B might compete with AMPK for binding to PP2A. The changes in interaction translated to altered AMPK phosphorylation (Figure 4A input). This phenomenon was further confirmed in CDC25B knockout or stable knockdown cells, where the AMPK–PP2A interaction was significantly increased when compared to that in control cells (Figure 4B). On the other hand, knockdown of AMPK resulted in an increased CDC25B–PP2A interaction (Figure 4C). Furthermore, inhibition of metformin-induced AMPK phosphorylation by CDC25B depletion could be rescued by the treatment of PP2A inhibitor LB100 (Figure 4D). Finally, knockdown of CDC25B decreased PP2A-C phosphorylation at Tyr-307 site (37) (Supplementary Figure S6A), and the phosphorylation led to an inactivated PP2A enzyme, indicating that CDC25B could regulate PP2A-C phosphorylation. However, this regulation was not direct, which was further confirmed by decreased PP2A phosphatase activity in the presence of GST-CDC25B (Supplementary Figure S6B). Thus, our data suggest that CDC25B controls AMPK activity via a mechanism that requires PP2A, in that overexpression of CDC25B can both decrease AMPK–PP2A interaction as well as reduce PP2A phosphatase activity.

### CDC25B–PP2A regulates metformin response

**Figure 5.**
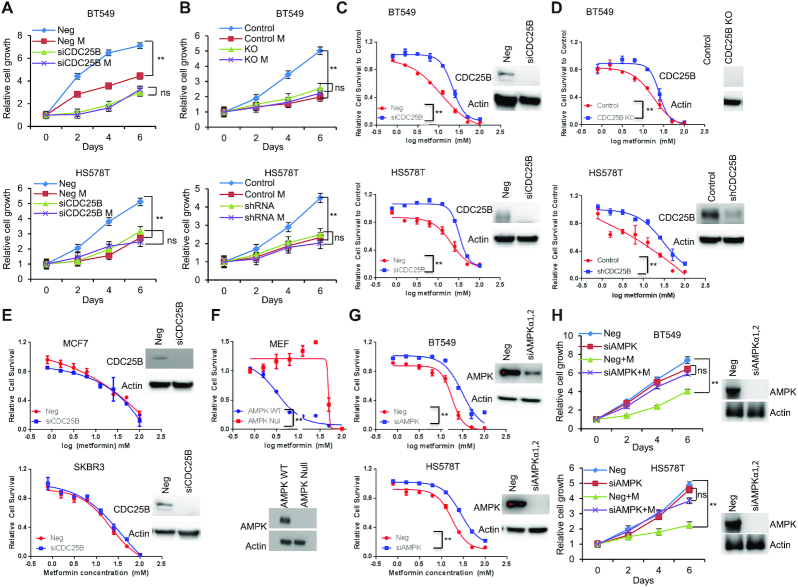
CDC25B modulation of metformin cytotoxicity is AMPK-dependent. (**A**) BT549 and HS578T cells were transfected with siCDC25B, and then treated with 20 mM metformin for 6 days. Cell proliferation was monitored every 2 days. The *x*-axis indicates time post treatment, and the *y*-axis indicates relative cell growth. (**B**) CDC25B knockout and stable knockdown BT549 and HS578T cells and their corresponding controls were treated with 20 mM metformin for 6 days. Cell proliferation was monitored every 2 days. The *x*-axis indicates time post treatment, and the *y*-axis indicates relative cell growth. (**C**) BT549 and HS578T cells transfected with siCDC25B were treated with increasing doses of metformin for 72 h, and cell survival was then determined. The *x*-axis indicates drug dose, and the *y*-axis indicates the survival fraction after metformin exposure. Knockdown efficiency is shown in western blots. (**D**) CDC25B knockout and stable knockdown BT549 and HS578T cells and their corresponding controls were treated with increasing doses of metformin for 72 h, and cell survival was then determined. The *x*-axis indicates drug dose, and the *y*-axis indicates the survival fraction after metformin exposure. Knockout efficiency is shown in western blots. (**E**) MCF7 and SKBR3 cells transfected with siCDC25B were treated with increasing doses of metformin for 72 h, and cell survival was then determined. The *x*-axis indicates drug dose, and the *y*-axis indicates the survival fraction after metformin exposure. Knockdown efficiency is shown in western blots. (**F**) AMPK null MEF and control MEFs were treated with increasing doses of metformin for 72 h, and cell survival was then determined. The *x*-axis indicates drug dose, and the *y*-axis indicates the survival fraction after metformin exposure. Knockdown efficiency is shown in western blots. (**G**) BT549 and HS578T cells transfected with siAMPK were treated with increasing doses of metformin for 72 h, and cell survival was then determined. The *x*-axis indicates drug dose, and the *y*-axis indicates the survival fraction after metformin exposure. Knockdown efficiency is shown in western blots. Error bars represent the ± SEM of three independent experiments. **P< 0.01. Statistical test: two-tailed *t*-test. (**H**) Cells were transfected with siAMPK, and then treated with 20 mM metformin for 6 days. Cell proliferation was monitored every 2 days. Data information: all data presented are in the format of mean ± SEM of *N* = 3 independent experiments with three biological replicates for each experiment. For (A, B and H), statistically significant differences were determined using the two-way ANOVA plus Tukey (**P < 0.01), ns represents not significant. For (C, D, E, F and G), statistically significant differences were determined using the two-way ANOVA (**P < 0.01).

We demonstrated that the CDC25B–PP2A interaction is important for AMPK activity. While the role of AMPK in metastasis is controversial (20–21,23,38), it is generally believed to be a tumor suppressor in triple negative breast cancer (39,40). Metformin, an AMPK activator, inhibited triple negative breast cancer cell growth (Figure 5A). However, metformin failed to inhibit cell growth upon CDC25B knockdown (Figure 5A). We further confirmed that metformin had no effect on the growth of CDC25B knockout or stable knockdown cells (Figure 5B), suggesting that CDC25B might impinge in metformin response. To test this hypothesis, we treated CDC25B knockdown cells with increasing doses of metformin. Strikingly, knockdown of CDC25B conferred resistance to metformin in triple negative breast cancer cells compared to negative siRNA transfected cells (Figure 5C). Consistently, CDC25B knockout or stable knockdown cells were more resistant to metformin treatment compared to control cells (Figure 5D). CDC25B, however, had no impact on metformin response in ER positive or HER2 positive breast cancer cells (Figure 5E). Since glucose concentration was found to influence metformin sensitivity in previous studies (41), we also tested CDC25B knockdown under low glucose condition and observed a similar resistance effect to metformin in triple negative breast cancer cells (Supplementary Figure S7). One of metformin's primary anticancer mechanisms is mediated through AMPK activation (42,43). We confirmed the role of AMPK in metformin response in our system. As shown in Figure 5F, AMPK-null MEFs were more resistant to metformin compared to AMPK WT MEFs, and this was also confirmed in triple negative breast cancer cells, where knockdown of AMPK decreased metformin sensitivity (Figure 5G). Furthermore, as shown in figure 5H, metformin had little effect on the growth of AMPK knockdown cells, suggesting that metformin's effect on cell growth is at least partially dependent on AMPK.

**Figure 6.**
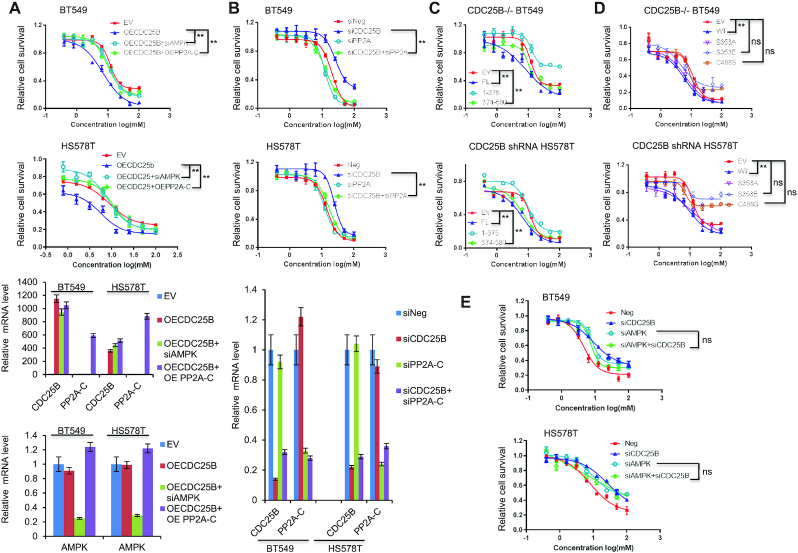
CDC25B modulation of metformin cytotoxicity via PP2A-AMPK. (**A**) BT549 and HS578T cells were transfected with EV, CDC25B or PP2A-C plasmids, negative siRNA or siAMPKα1 and α2, and then treated with increasing doses of metformin for 72 h, and cell survival was then determined. The *x*-axis indicates drug dose, and the *y*-axis indicates the survival fraction after metformin exposure. Knockdown or overexpression efficiency is shown in mRNA levels. (**B**) CDC25B knockdown BT549 and HS578T were transfected with siPP2A, and then treated with increasing doses of metformin for 72 h, and cell survival was then determined. The *x*-axis indicates drug dose, and the *y*-axis indicates the survival fraction after metformin exposure. Knockdown efficiency is shown in mRNA levels. (**C**) CDC25B knockout BT549 and stable knockdown HS578T cells transfected with EV, full length CDC25B (FL), CDC25B N-terminal domain (1–375 aa) (NTD), or CDC25B C-terminal domain (374–580 aa) (CTD), were treated with increasing doses of metformin for 72 h and cell survival was then determined. The *x*-axis indicates drug dose, and the *y*-axis indicates the survival fraction after metformin exposure. (**D**) CDC25B knockout BT549 and stable knockdown HS578T cells transfected with EV, WT CDC25B (WT), CDC25B S353A, CDC25B S353E, or CDC25B C488S constructs, were treated with increasing doses of metformin for 72 h and cell survival was then determined. The *x*-axis indicates drug dose, and the *y*-axis indicates the survival fraction after metformin exposure. (**E**) Cells were transfected with indicated siRNA, and then treated with increasing doses of metformin for 72 h, and cell survival was then determined. The *x*-axis indicates drug dose, and the *y*-axis indicates the survival fraction after metformin exposure. Data information: all data presented are in the format of mean ± SEM of *N* = 3 independent experiments with three biological replicates for each experiment. Statistically significant differences were determined using the two-way ANOVA plus Tukey (**P < 0.01), ns represents not significant.

Next, we tested whether CDC25B regulates metformin cytotoxicity through AMPK. To accomplish this, we overexpressed CDC25B in HS578T and BT549 cells with AMPK knockdown or PP2A-C overexpression. As shown in Figure 6A, CDC25B overexpression alone conferred increased sensitivity to metformin. AMPK knockdown reversed the sensitivity induced by CDC25B overexpression, indicating that CDC25B’s effect on metformin sensitivity is dependent on AMPK. To confirm the CDC25B–PP2A interaction has a functional impact, we performed dual overexpression of PP2A-C and CDC25B. Overexpression of both PP2A-C and CDC25B caused increased metformin resistance compared with CDC25B overexpression alone (Figure 6A). As previously observed, CDC25B knockdown decreased metformin sensitivity, but double knockdown of both CDC25B and PP2A-C reversed the resistance caused by CDC25B single knockdown and restored sensitivity to metformin (Figure 6B). Because the NTD, S353E and C488S mutations disrupt the CDC25B interaction with the C subunit of PP2A, we hypothesized that the NTD, S353E and C488S mutants might lose their impact on metformin response. Indeed, cells overexpressing NTD did not confer metformin sensitization compared to the FL or the CTD (Figure 6C). Overexpression of the S353E and C488S mutants in CDC25B knockout and stable knockdown cells also failed to sensitize cells to metformin compared to the WT CDC25B (Figure 6D). These results indicate that CDC25B regulation of metformin sensitivity is also dependent on PP2A-C’s regulation on AMPK. Indeed, double knockdown of both CDC25B and AMPK did not further increase metformin resistance compared with either single knockdown (Figure 6E).

### CDC25B–PP2A regulates breast cancer patients derived 3D organoids’ response to metformin

**Figure 7.**
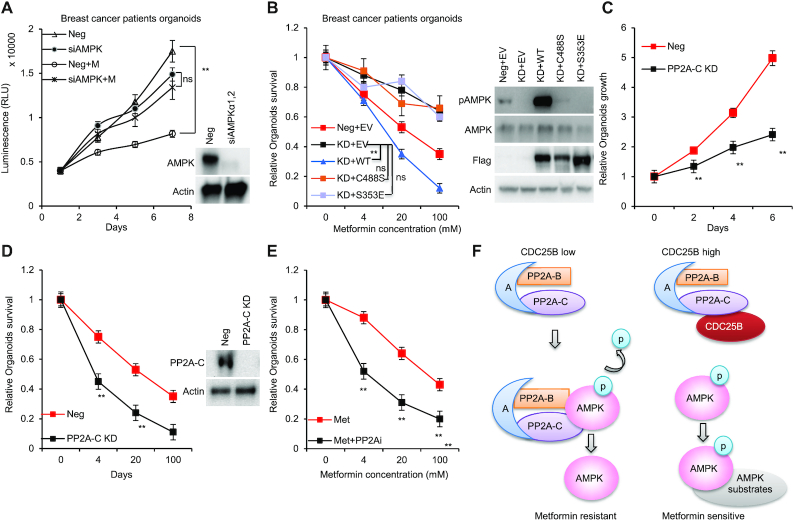
CDC25B–PP2A regulates breast cancer patients derived 3D organoids’ response to metformin. (**A**) Organoids transfected with siAMPK, and then treated with 20 mM metformin for 6 days. Cell proliferation was monitored every 2 days. (**B**) Organoids transfected with siCDC25B (KD) were transfected with EV, WT CDC25B (WT), CDC25B S353E or CDC25B C488S constructs, and then treated with increasing doses of metformin for 72 h, and organoid survival was then determined. The *x*-axis indicates drug dose, and the *y*-axis indicates the survival fraction after metformin exposure. Organoid lysates were blotted with the indicated antibodies. (**C**) Organoids were transfected with siPP2A-C, and the growth was monitored every 2 days. (**D**) Organoids transfected with siPP2A-C were treated with increasing doses of metformin for 72 h, and organoid survival was then determined. The *x*-axis indicates drug dose, and the *y*-axis indicates the survival fraction after metformin exposure. Knockdown efficiency is shown in western blots. (**E**) Organoids were treated increasing doses of metformin alone or in combination with 2 μM of LB100 for 72 h, and organoid survival was then determined. The *x*-axis indicates drug dose, and the *y*-axis indicates the survival fraction after metformin exposure. (**F**) A simplified model depicting how CDC25B might regulate AMPK activity. Data information: all data presented are in the format of mean ± SEM of *N* = 3 independent experiments with three biological replicates for each experiment. For (A and B), statistically significant differences were determined using the two-way ANOVA plus Tukey (**P < 0.01), ns represents not significant. For (C, D and E), statistically significant differences were determined using the two-way ANOVA (**P < 0.01).

To investigate the role of CDC25B–PP2A on metformin response in patients’ relevant model, we grew organoids from primary triple negative breast cancer patients’ derived xenograft (PDX) tumors (33). We first confirmed the role of AMPK in metformin-mediated growth inhibition. As shown in Figure 7A, metformin had little effect on the growth of AMPK knockdown organoids. We then knocked down endogenous CDC25B (KD organoids), and further determined metformin response by transfecting organoids with Neg + EV, CDC25B KD + EV, KD + Flag-CDC25B WT, KD + Flag-CDC25B C488S or Flag-CDC25B S353E constructs. Consistent with previous work, CDC25B KD decreased metformin sensitivity. Strikingly, metformin sensitivity was restored in CDC25B KD cells by overexpressing Flag-CDC25B WT, but not Flag-CDC25B C488S or S353E (Figure 7B, left panel). As expected, a higher level of phosphorylated AMPK was found in organoids transfected with Neg + EV compared to KD + EV and higher pAMPK in CDC25B KD organoids after overexpressing WT compared to the two mutants, CDC25B C488S and S353E as well as control cells (Figure 7B, right panel). Furthermore, when compared to the control group, PP2A-C knockdown reduced organoid growth (Figure 7C) and increased metformin sensitivity (Figure 7D), suggesting that PP2A might promote organoid growth. Furthermore, the PP2A inhibitor, LB100, sensitized the breast cancer organoids to metformin treatment (Figure 7E and Supplementary Figure S8). Collectively, these data indicate that the CDC25B is essential for its role in metformin response via its interaction with PP2A.

## DISCUSSION

CDC25 proteins are generally known as dual phosphatase specific protein that dephosphorylate and activate CDK activity during cell cycle progression (44,45). CDC25 proteins are key targets of the checkpoint machinery to maintain genome stability during DNA damage (46–48). CDC25A and B expression has been shown to be stimulated by the c-MYC oncogene and has been reported in many types of human cancers (1,7), possibly resulting from genome instability caused by the checkpoint-abrogating effect of their overexpression. However, CDC25 may contribute to tumorigenesis by other mechanism. Herein, we uncovered a novel function of CDC25B. It served as a regulator for AMPK activity mediated by its interaction with PP2A, the prototypic Ser/Thr phosphatase in cells (Figure 7F).

Our findings in this paper have underscored the observation that CDC25B is a protein with diverse functions fulfilled by its different domains. The CTD of CDC25B contains the phosphatase activity and interacts with PP2A. The NTD of CDC25B can be phosphorylated by JNK and p38, resulting in rapid degradation of CDC25B (49), and the binding of 14–3-3 proteins and nuclear export regulate the intracellular localization of CDC25B (50). CDC25B mediates the rapamycin-induced activation of AKT, elF4E and p38 cascades, and CDC25B knockdown promotes the inhibitory effect of rapamycin on cancer cells growth through blocking cell cycle progression and inhibiting oncogenic pathways activation by rapamycin (51). In this paper, we demonstrated that the CDC25B–PP2A interaction played a critical role in mediating AMPK activation and metformin response in triple negative breast cancer (Figure 6). It appeared that CDC25B depletion could also inhibit 2-deoxyglucose and oligomycin- induced AMPK activation, but not A7696620-induced AMPK activation (Supplementary Figure S10A). A769662 is a direct AMPK activator, which binds to AMPK at sites distinct from the AMP sites. These results suggest that CDC25B effect on AMPK activity is resulted from the alteration in cellular AMP/ATP changes induced by metformin. However, it did not act on the entry of metformin into the cells as it did not alter the level of metformin transporter (Supplementary Figure S10B). It has been shown that a spatiotemporal regulation of AMPK complexes exist in different compartments by both AMP-dependent and AMP-independent mechanisms (52). There may be more than one mechanism to regulate AMPK activity in cells depending on the degree of stress severity or the stimulus (52). The use of direct AMPK activator may help to overcome metformin resistance in triple negative breast cancer patient. In our study, we have used relatively high metformin concentrations. While the underlying cause remains a point of debate, it has been established that cells grown in culture require much higher concentrations of metformin to elicit responses that are similar to those seen *in vivo* (53).

CDC25B has been found to be associated with metformin response in our previous pharmacogenomic study (54) using 266 lymphoblastoid cell lines (Supplementary Figure S9). Given AMPK’s multiple roles in tumor (55), CDC25B may contribute to various behaviors of tumor cells, at least in part through controlling AMPK activity via CDC25B-PP2A association. Moreover, as the PP2A phosphatase has been reported to modulate anticancer immunity (56), our finding of the CDC25B–PP2A interaction might impact tumor progression not only through regulating AMPK activity, but also through immune modulation. Finally, because the interaction with PP2A occurred in the CTD of CDC25B (Figure 2F), which also contains the phosphatase activity of CDC25B, therefore, it is possible that this association could affect CDC25B phosphatase functions.

Our results also have revealed CDC25B as a previously unrecognized regulator of PP2A, a major Ser/Thr phosphatase in cells. It is also worth noting that a previous report suggests that PP2A is typically regulated through its B subunits, which determine the substrate specificity, subcellular localization and enzymatic activity of the holoenzyme (57). However, under our experimental conditions, the C subunit, but not the B subunit, of PP2A had a direct interaction with CDC25B (Figure 3). Thus, the binding of PP2A to CDC25B proteins via C subunit may result in altered PP2A phosphatase activity. Indeed, we demonstrated that CDC25B bound to PP2A specifically through its C subunit in both cytoplasm and nucleus (Supplementary Figure S10C), with the B56δ subunit in the complex, leading to a decreased PP2A phosphatase activity and increased breast cancer cell growth. This observation is consistent with two recent studies showing that the PP2A contributed to tumor progression through enhancing target gene occupancy of c-Jun and stimulating oncogenic signaling (ERK, AKT and WNT) in colorectal and pancreatic cancer models (58,59). Thus, although PP2A is considered to be a tumor suppressor, it may stimulate tumor progression through additional mechanisms such as, in this case, binding to CDC25B. CDC25B and CDC25A can compensate for each other in some of their functions (60); however, *in vitro* study combining both CDC25B and CDC25A did not further inhibit PP2A activity (Supplementary Figure S10D). Simultaneous knocking down of CDC25A and CDC25B did not further inhibit metformin induced AMPK activation, and overexpression of CDC25A in CDC25B-depleted cells did not rescue AMPK activation (Supplementary Figure S10E).

Finally, our results revealed a mechanism of regulating the activity of a major energy sensor and modulator of cell growth and metabolism, AMPK (Figure 1 A–C). AMPK activation has been suggested to have tumor-suppressive properties in some contexts and may mediate some of the cell-autonomous benefits of mitochondrial inhibitors on tumor cell growth, although AMPK activation may also allow tumor cell survival under the metabolic stress conditions that many tumor cells face. Previous studies have shown that PP2A dephosphorylates Thr-172 and reduces AMPK activity via physically interacting with AMPK through its B55δ subunit (61). Here, we demonstrated that CDC25B, by binding to PP2A containing B56δ but not B55δ subunit, led to increased phosphorylation of Thr-172 and increased AMPK activity (Figure 3). Our data showed that CDC25B knockdown increased AMPK–PP2A interaction (Figure 4A and B) indicating that the regulation of AMPK activity by CDC25B is dependent on PP2A. Even though our data did not provide evidence that CDC25B directly dephosphorylated PP2A, previous study has shown that PP2A associates with CDC55 and dephosphorylates Mih1, the budding yeast homolog of CDC25 (62). Further, our organoids’ experiments demonstrated that AMPK activity was regulated by CDC25B, as the AMPK activity was higher in CDC25B KD cells when compared to the control cells or KD cells re-expressing WT CDC25B (Figure 7B). Taken together, these data suggest that CDC25B is involved in the regulation of AMPK activity, and this regulation might depend on the participation of different regulatory B subunits and the availability of PP2A. We did not observe the interaction with B55δ subunit of PP2A which was reported previously. One reason might be due to different tissue or cell-specific expression of the subunits. Interestingly, we did not identify a direct dephosphorylation site of PP2A by CDC25B, indicating that CDC25B indirectly regulates PP2A-C phosphorylation at Tyr-307 and its phosphatase activity. PP2A enzymes can be inactivated following tyrosine phosphorylation of the catalytic subunit at the putative Tyr-307 site, via activation of SRC kinase, epidermal growth factor receptor or insulin signaling (37). Other modifications of PP2A-C subunit include tyrosine nitration that increases PP2A activity in endothelial cells (63). Adding another layer of complexity to the regulation of PP2A holoenzymes, protein kinase A-mediated serine phosphorylation of selective B55α and B55δ regulatory subunits can also modulate PP2A catalytic activity (64,65). Whether CDC25B regulates upstream enzymes responsible for PP2A-C phosphorylation or nitration, or whether CDC25B is involved in regulating the phosphorylation of selective B regulatory subunits, regulating AMPK activity, remains to be elucidated.

It is well established that AMPK plays a pivotal role in maintaining energy and redox homeostasis under various metabolic stress conditions. While it is known that activation of this energy sensor inhibits cancer cell proliferation and growth through inhibition of anabolic processes, AMPK activation can confer cancer cell plasticity to survive under metabolic stress such as hypoxia and glucose deprivation, which are commonly observed in fast growing tumors. Therefore, the tumor-suppressing or tumor-promoting roles of AMPK depend on the cell or tissue context. Understanding the molecular mechanism of how AMPK activity is regulated (such as by CDC25B and PP2A) provides better understanding the roles of AMPK in cancer development, progression as well as response to treatment.

## DATA AVAILABILITY

The Mass Spectrometry data have been deposited in a repository affiliated with the ProteomeX consortium.

Project Name: Identify proteins that interact with CDC25B Project accession: PXD020368

## Supplementary Material

zcaa039_Supplemental_File
