# Supplementary material for: CDC25B partners with PP2A to induce AMPK activation and tumor suppression in triple negative breast cancer
Source: NAR Cancer. 2020 Dec 21;2(4):zcaa039. doi: 10.1093/narcan/zcaa039 (PMC7751685; doi:10.1093/narcan/zcaa039)

SUPPLEMENTARY DATA

Cell cycle analysis

After treatment of 20 mM metformin for 24 hours, cells were trypsinized and washed by phosphate-buffered saline (PBS). After fixation in ice-cold 70% ethanol overnight at 4 °C, cells were washed with PBS containing 1% BSA, and then resuspended in PBS containing 1% BSA, 20 μg/ml propidium iodine, and 100μg/ml RNase A. Flow cytometry assay was performed with a FACSCalibur cytometer (BD Biosciences) and 50000 cells were collected for data analysis. The percentage of cells in each phase of the cell cycle was estimated with ModFit.

Cell cycle synchronization

Cells were grown to 60% confluence, and treated with thymidine for 18 hrs in tissue culture incubator. After 18 hrs, remove thymidine by washing with 1X PBS and adding fresh media, and incubate for 9 hrs. Repeat the 2 mM of thymidine incubation for an additional 14 hours. Remove the media and wash culture with PBS. Add fresh culture media. At this point, cells were at G1/S phase and are ready to be released into cycle.

Metformin genome-wide association studies (GWAS) using LCLs

As described in our previous publication (1), EBV-transformed LCLs from 96 African-American (AA), 96 Caucasian-American (CA), and 96 Han Chinese-American (HCA) unrelated subjects were purchased from the Coriell Cell Repository (Camden, NJ). These samples had been anonymized by NIGMS, and all subjects had provided written consent for their experimental use. This study was reviewed and approved by Mayo Clinic Institutional Review Board. As described previously (1), we genotyped DNA samples from the LCLs in the Genotype Shared Resource (GSR) at Mayo Clinic using Illumina HumanHap 550K and 510S BeadArrays, which contained 561,298 and 493,750 SNPs respectively. Publicly available Affymetrix SNP Array 6.0 Chip data for additional 643,600 SNPs were also obtained for the same cell lines. All the genotyping data are used in our previous studies (1) and is publicly available from NCBI Gene Expression Omnibus (http://www.ncbi.nlm.nih.gov/geo) under SuperSeries accession No. GSE24277. SNPs that deviated from Hardy-Weinberg Equilibrium (HWE) based on the minimum p-value from an exact test for HWE and the stratified test for HWE (p-values <0.001); SNPs with call rates <95%; or SNPs with minor allele frequencies (MAFs) <5% were removed from the analysis. Total RNA was extracted from each of the cell lines using Qiagen RNeasy Mini kits (QIAGEN, Inc.). RNA quality was tested using an Agilent 2100 Bioanalyzer, followed by hybridization to Affymetrix U133 Plus 2.0 Gene-Chips. The expression array data for all 54k probe sets was used in our previous studies (1) and is publicly available from NCBI Gene Expression Omnibus (http://www.ncbi.nlm.nih.gov/geo) under SuperSeries accession no. GSE24277 and accession No.GSE23120.

The metformin cytotoxicity phenotype IC50, indicating the drug concentration that inhibits half of maximal cell growth, was calculated based on a logistic model. Three different logistic functions (a four parameter logistic model, a three parameter logistic model with a fixed asymptote at 0%, and a three parameter logistic model with a fixed asymptote at 100%) were used to fit the data with the R package ‘‘drc’’ (http://cran.r-project.org/web/packages/drc/drc.pdf) (1). The best model fit (i.e., lowest mean square error) from the three logistic models was used to determine the cytotoxicity IC50 phenotype. Prior to association analyses, the Van der Waerden (rank) transformed IC50 and SNPs were adjusted for gender, race and population stratification. Similarly, GCRMA normalized and log2 transformed mRNA expression array data were adjusted for gender, race, population stratification, as well as batch effect (1). For DNA methylation data, the logit of beta values were adjusted for age, gender and race using linear regression model, followed by adding overall mean probe values back followed by back transformation of the values using inverse logit function. The final adjusted beta values on the [0,1] scale were used for analysis (1).

Pair wised association among adjusted SNP, expression, methylation and IC50 values were completed using Pearson correlations. False Discovery Q-values were also computed for each test. Genes and SNPs were annotated using NCBI Build 37 (1). We first identified top SNP loci or methylation probes/loci associated with IC50, then determined which expression probe sets were associated with these SNPs/methylation probes, and finally determined whether the expression probe sets associated with these SNPs/methylation probes were also associated with metformin IC50 values.

REFERENCES

1. Niu, N., Liu, T., Cairns, J., Ly, R.C., Tan, X., Deng, M., Fridley, B.L., Kalari, K.R., Abo, R.P., Jenkins, G. *et al.* (2016) Metformin pharmacogenomics: a genome-wide association study to identify genetic and epigenetic biomarkers involved in metformin anticancer response using human lymphoblastoid cell lines. *Hum Mol Genet*, **25**, 4819-4834.

FIGURES LEGEND

Supplementary Figure S1. CDC25B and cell cycle. (A) HS578T cells were transfected with negative control and CDC25B siRNA. Cell cycle profile was analyzed by flow cytometry. (B) BT549 and HS578T cells were transfected with indicated siRNA. Cell proliferation was monitored every 2 days. The x-axis indicates time post transfection, and the y-axis indicates relative cell growth. Knockdown efficiency was shown in western blot. Data information: all data presented are in the format of mean ± SEM of N=3 independent experiments with 3 biological replicates for each experiment. Statistically significant differences were determined using two-way ANOVA plus Tukey (**p < 0.01).

Supplementary Figure S2. (A) CDC25B and PP2A interaction at different cell cycle phases. BT549 and HS578T cells were synchronized by double thymidine block. Cells were harvested at 0, 4, 8, 12, 18, and 24 hours after release. Cell lysates were subjected to immunoprecipitation with control IgG or anti- CDC25B antibody. The immunoprecipitates were blotted with the indicated antibodies. (B) PP2A phosphatase activity affects the binding between CDC25B and PP2A. HS578T and BT549 cells were treated with 2 μM of PP2A inhibitor, LB100, for 24 hours, cell lysates were subjected to immunoprecipitation with control IgG or anti- CDC25B antibody. The immunoprecipitates were blotted with the indicated antibodies. Data information: all data presented are a representation of N=3 independent experiments.

Supplementary Figure S3. Thr and Ser phosphatase activity of PP2A-B56δ. The PP2A complex was assembled using equal amount of purified His-A, His-B56δ and His-C prior to evaluating its Thr and Ser Phosphatase activity. pThr or pSer peptide substrates were incubated with 200–500 ng of PP2A protein. Malachite Green mixture was used to measure absorbance at 620 nm. All phosphatase activity assays were normalized to the amount of protein used. The PP2A complex has both Thr and Ser Phosphatase activity. Data information: all data presented are in the format of mean ± SEM of N=3 independent experiments with 3 biological replicates for each experiment. Statistically significant differences were determined using two-way ANOVA plus Tukey (**$p < 0.01$).

Supplementary Figure S4. CDC25B does not interact with LKB1 or CaMKKβ. (**A**) HEK-293T cells were transfected with empty vector (EV) or Flag-CDC25B plasmids for 48 hours. Cell lysates were subjected to immunoprecipitation with anti-Flag antibody. The immunoprecipitates were blotted with the indicated antibodies. (**B**) BT549 and HS578T cell lysates were subjected to immunoprecipitation with control IgG or anti- CDC25B antibody. The immunoprecipitates were blotted with the indicated antibodies. Data information: all data presented are a representation of N=3 independent experiments.

Supplementary Figure S5. Depletion of CDC25B does not change the AMPK - LKB1 interaction or AMPK - CaMKKβ interaction. HS578T cells were transfected with Negative siRNA or siCDC25B. 48 hours later, cell lysates were subjected to immunoprecipitation with control IgG or anti-AMPK antibody. The immunoprecipitates were blotted with the indicated antibodies. Data information: all data presented are a representation of N=3 independent experiments.

Supplementary Figure S6. CDC25B decreases PP2A phosphatase activity. (**A**) HS578T breast cancer cells were transiently transfected with negative siRNA (Neg) or CDC25B siRNA for 48 hours. Cells were harvested for immunoblotting with the indicated antibodies. (**B**) HS578T cell lysates were incubated with PP2A-C antibody. Malachite green was used to measure absorbance of 650 nm. Phosphatase activity was determined using a standard curve. Data information: all data presented are in the format of mean ± SEM of N=3 independent experiments with 3 biological replicates for each experiment. Statistically significant differences were determined using two-way ANOVA (**$p < 0.01$).

Supplementary Figure S7. CDC25B knockdown confers resistance to metformin under low glucose conditions. HS578T cells were transiently transfected with CDC25B siRNA for 24 hours in

DMEM/10% FBS (high glucose). Afterwards, media was replaced for MEM/10% FBS (low glucose) followed by 48 hours metformin treatment. Cell survival was then determined. The x-axis indicates drug dose, and the y-axis indicates the survival fraction after metformin exposure. Knockdown efficiency is shown in qRT-PCR. Data information: all data presented are in the format of mean ± SEM of N=3 independent experiments with 3 biological replicates for each experiment. Statistically significant differences were determined using two-way ANOVA (**p < 0.01).

Supplementary Figure S8. PP2A inhibitor sensitized triple negative breast cancer cells to metformin treatment. BT549 and HS578T cells were treated with increasing doses of metformin alone, or in combination with PP2A inhibitor (PP2Ai) for 72 hours, and cell survival was then determined. The x-axis indicates metformin dose, and the y-axis indicates the survival fraction after treatment exposure. Data information: all data presented are in the format of mean ± SEM of N=3 independent experiments with 3 biological replicates for each experiment. Statistically significant differences were determined using two-way ANOVA (**p < 0.01).

Supplementary Figure S9. CDC25B is associated with metformin sensitivity in a GWAS using 266 lymphoblastoid cell lines (LCLs) treated with metformin. Metformin genome-wide association analyses with mRNA expression in the LCLs. The y-axis represents the –log10 (p-value) for the association of individual expression array probe set. The data are plotted on the x-axis based on the chromosomal locations of genes.

Supplementary Figure S10. CDC25B inhibits AMPK activation via PP2A. (A) BT549 and HS578T cells were transfected with negative siRNA (Neg) or CDC25B siRNA for 24 hours, and then treated with indicated treatments: 400µM A769662 for 24 hours, 25mM 2-DG for 24 hours, 1µM oligomycin for 30 minutes. Cells were harvested for immunoblotting of AMPK activation. (B) BT549 and HS578T cells were transfected with negative siRNA (Neg) or CDC25B siRNA for 24 hours, mRNA were harvested for qRT-PCT to detect metformin transporter, OCT1, OCT2, and OCT3. Only OCT1 was detectable in breast cancer cells. (C) BT549 and HS578T cell lysate nuclear and cytoplasmic fraction were subjected to immunoprecipitation with control IgG or anti-CDC25B antibody. The immunoprecipitates were blotted with the indicated antibodies. (D) HS578T cell lysates were incubated with PP2A-C antibody. The immunoprecipitates were incubated with GST-tagged proteins. Malachite green was used to measure absorbance of 650 nm. Phosphatase activity was determined using a standard curve. (E) HS578T cells were transfected with indicated siRNA and plasmid for 24 hours, and then treated with 20 mM metformin for 48 hours. Cells were harvested for immunoblotting of AMPK activation. Data information: all data presented are in the format of mean ± SEM of N=3 independent experiments with 3 biological replicates for each experiment. For (B), statistically significant differences were determined using two-way ANOVA and there is no difference between Neg and siCDC25B (**p < 0.01). For (D), statistically significant differences were determined using two-way ANOVA plus Tukey (**p < 0.01), ns represents not significant.

Supplementary Figure S1

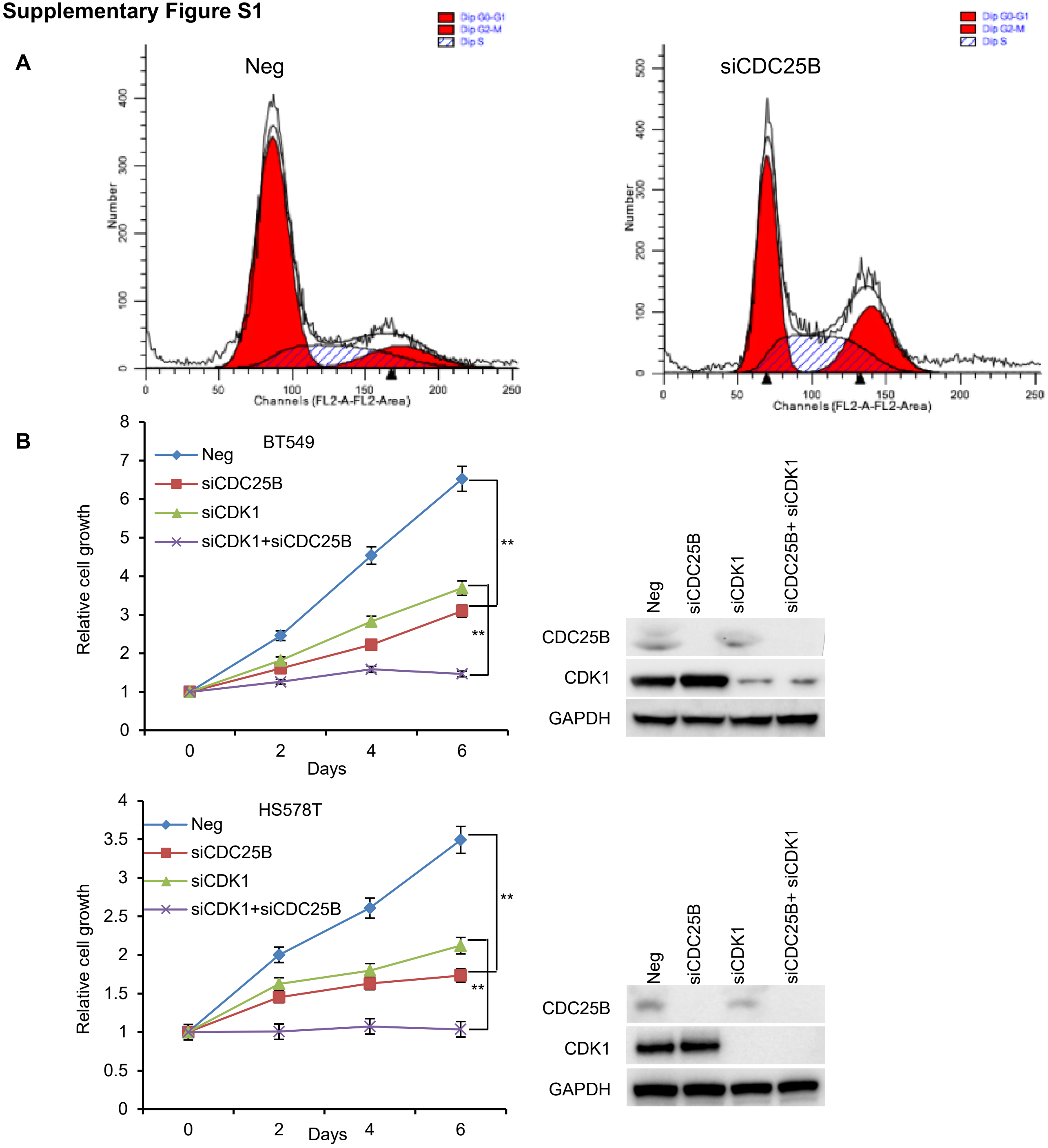

Supplementary Figure S2

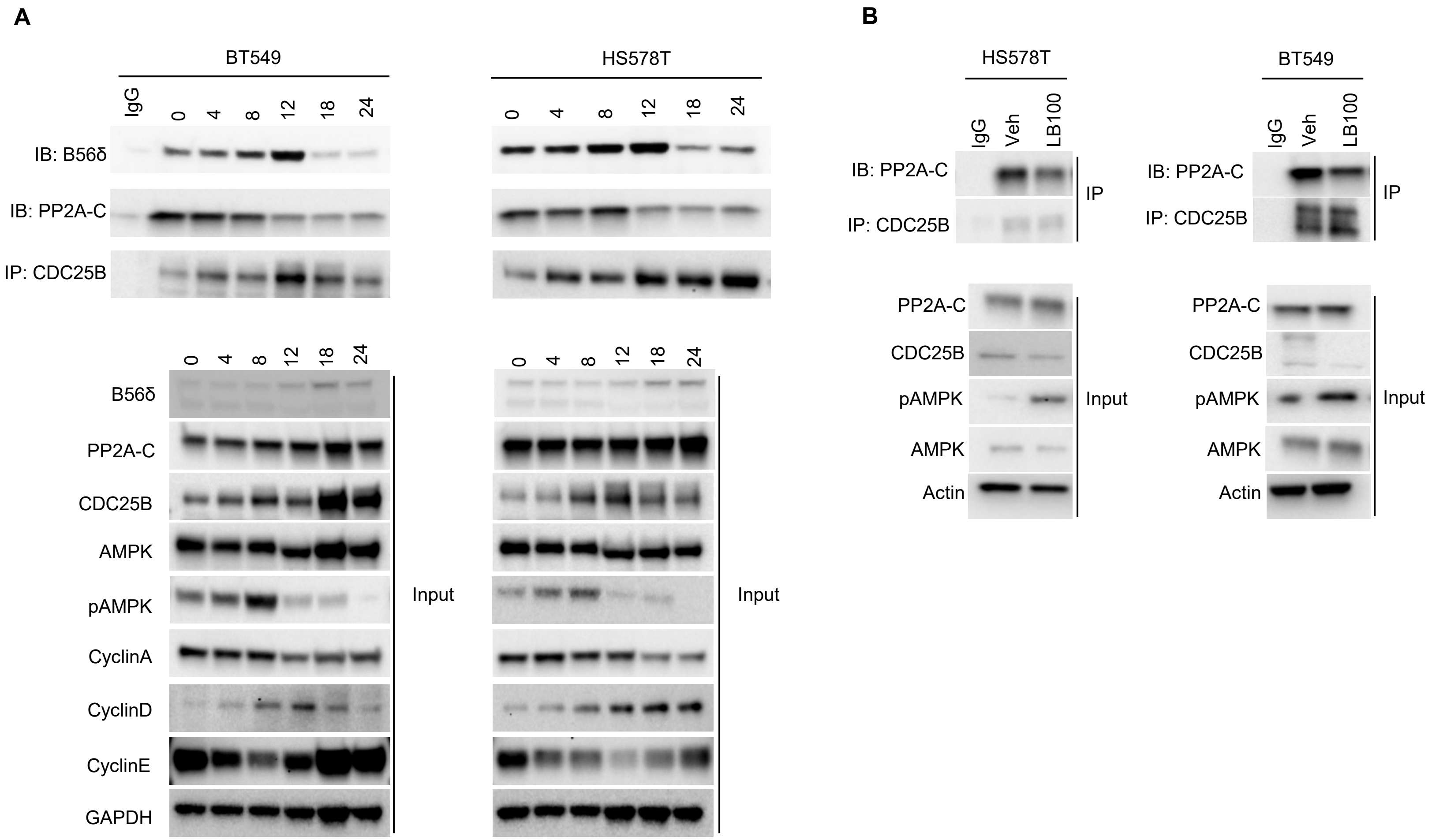

Supplementary Figure S3

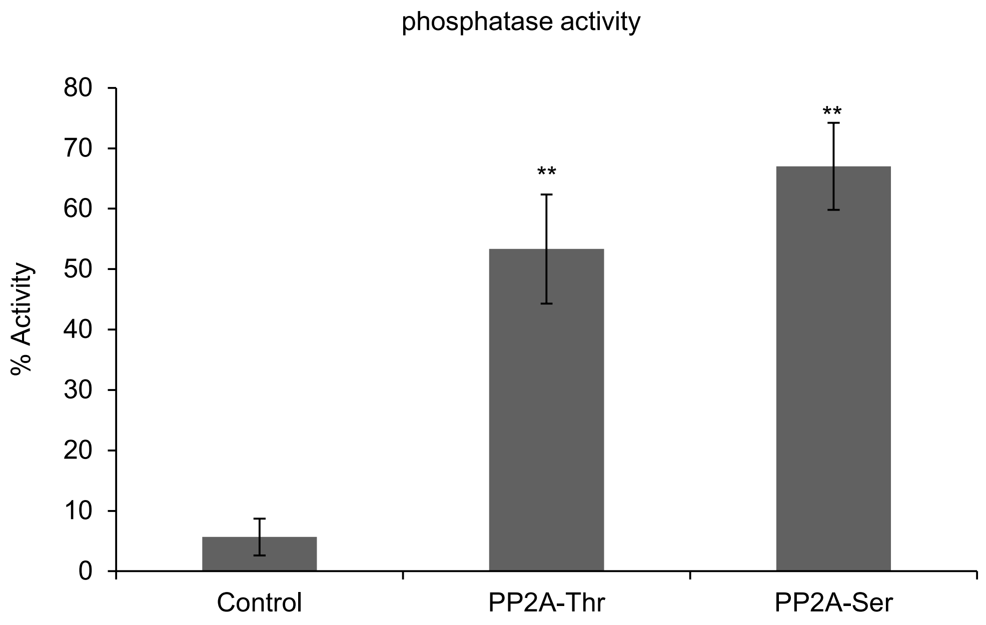

Supplementary Figure S4

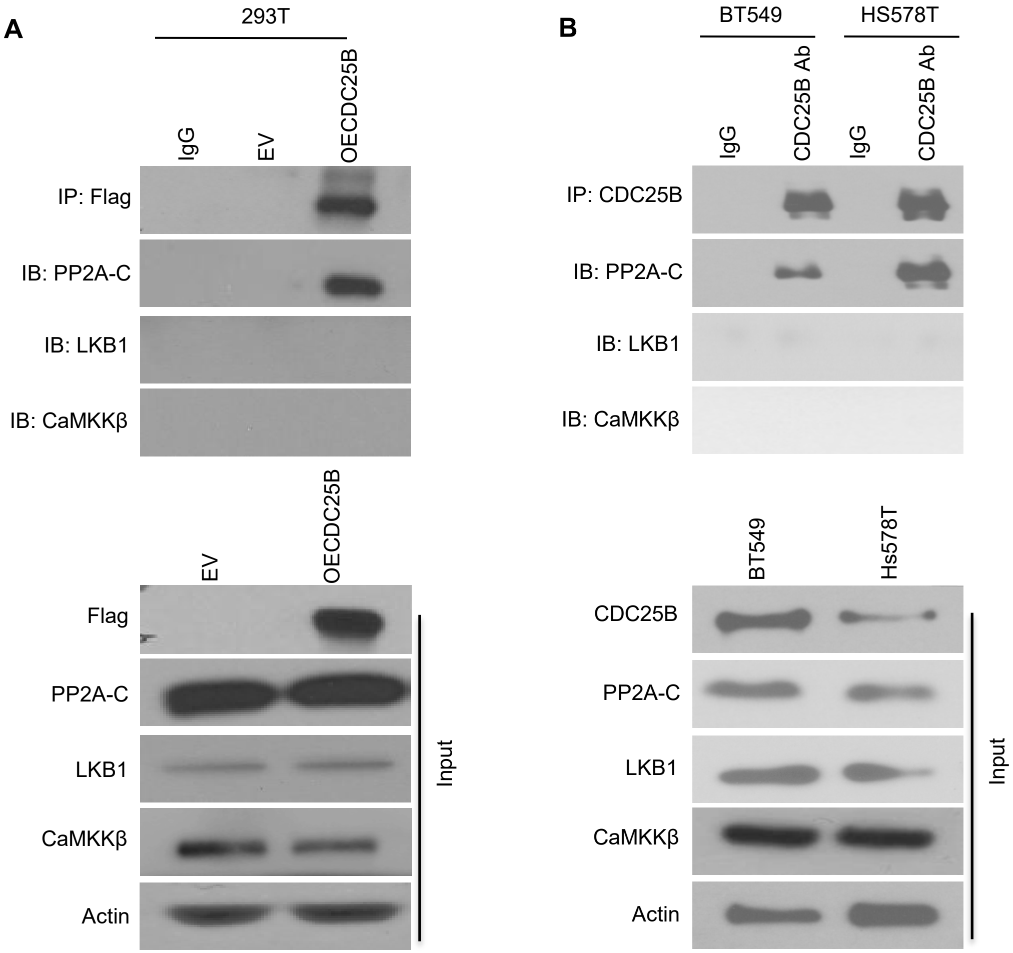

Supplementary Figure S5

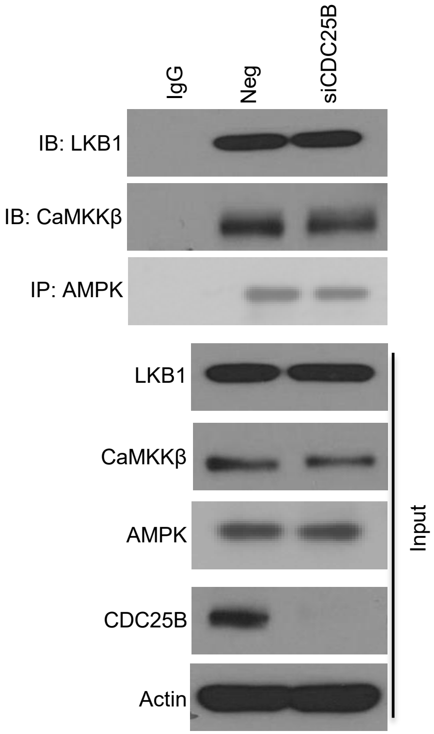

Supplementary Figure S6

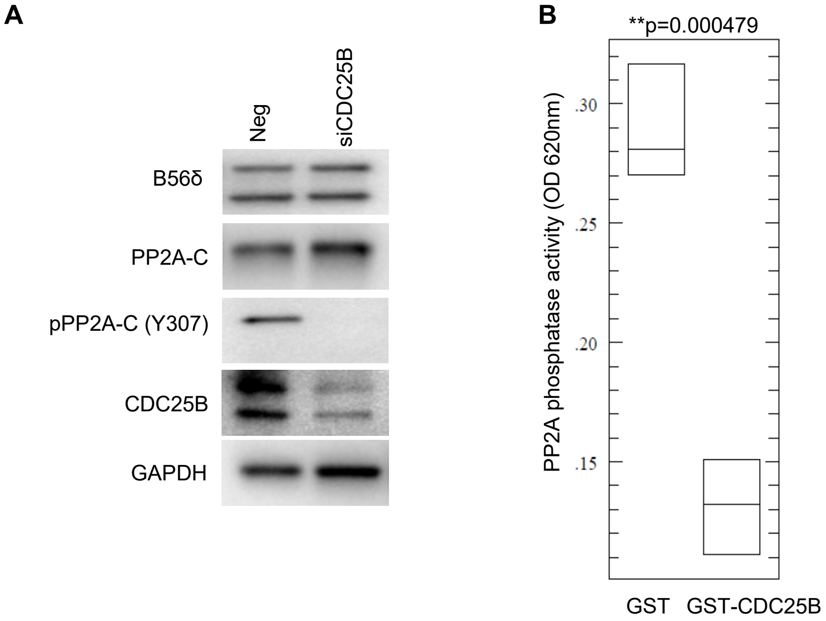

Supplementary Figure S7

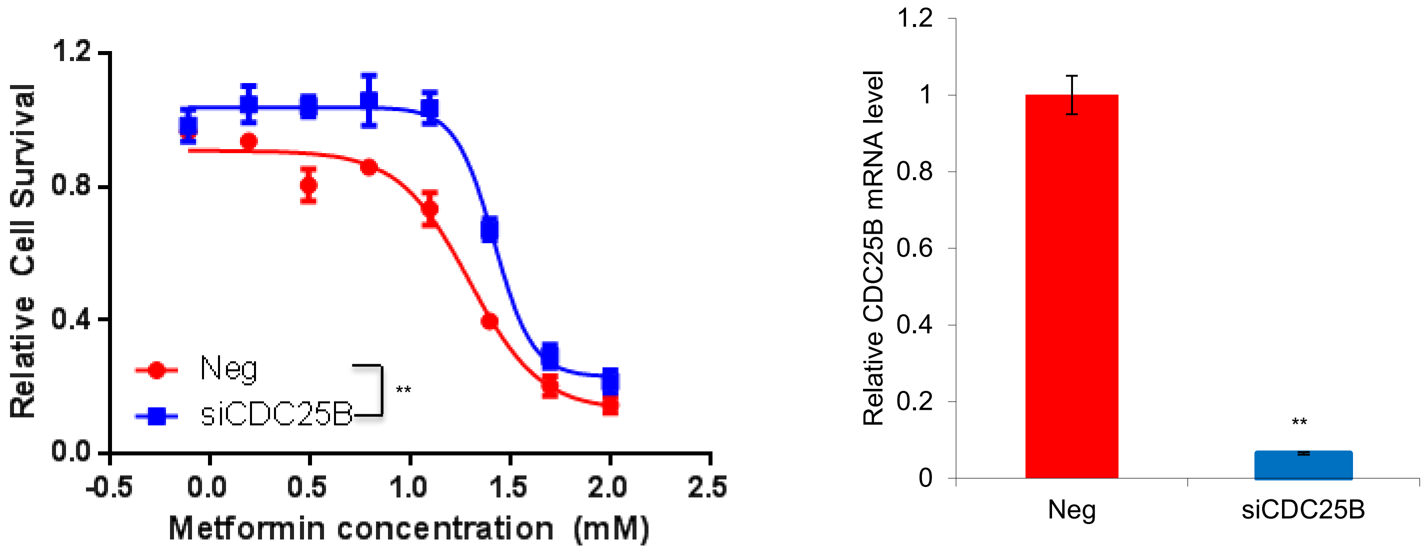

Supplementary Figure S8

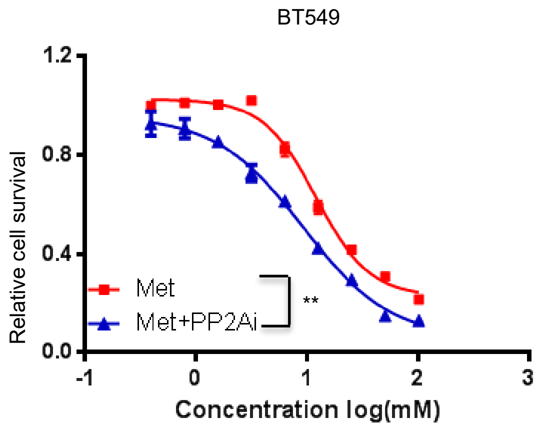

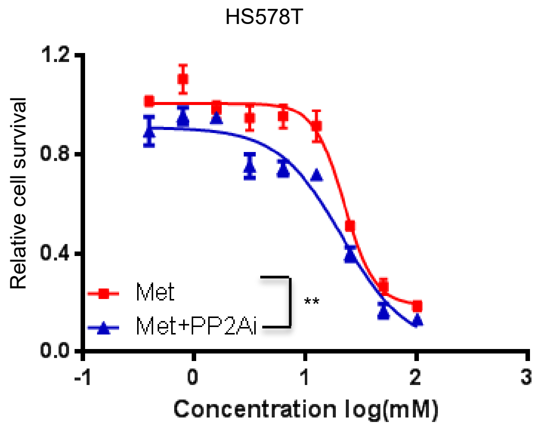

Supplementary Figure S9

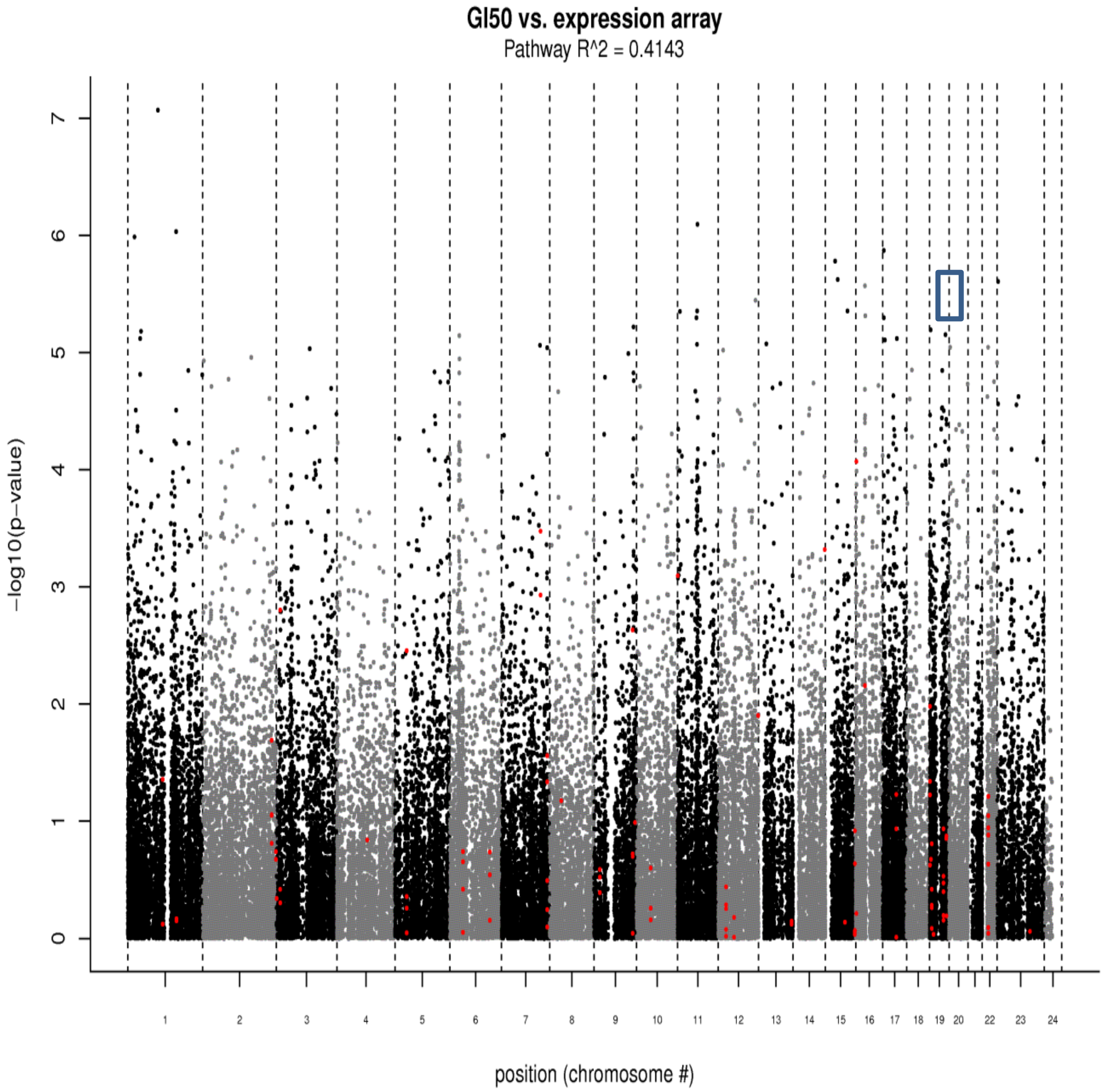

Probe ID	P value	R value	Q value	Chr	Gene Symbol
201853_s_at	9.26E-06	-0.267	0.013	20	CDC25B

Supplementary Figure S10

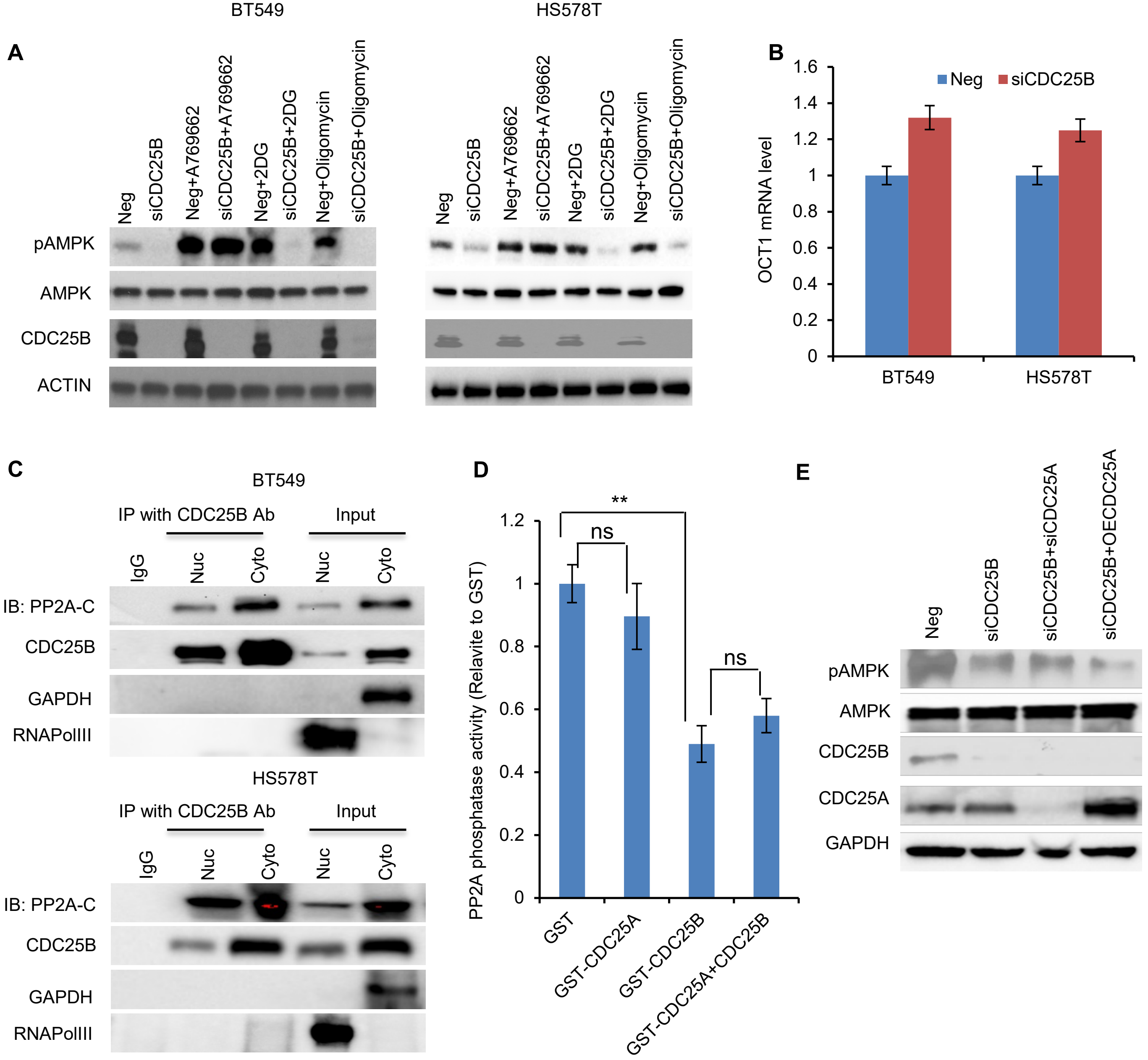

Supplement: zcaa039_Supplemental_File [file zcaa039_supplemental_file.pdf]
